# Progranulin promotes peripheral nerve regeneration and reinnervation: role of notch signaling

**DOI:** 10.1186/s13024-016-0132-1

**Published:** 2016-10-22

**Authors:** Christine Altmann, Verica Vasic, Stefanie Hardt, Juliana Heidler, Annett Häussler, Ilka Wittig, Mirko H. H. Schmidt, Irmgard Tegeder

**Affiliations:** 1 Institute of Clinical Pharmacology, Goethe-University Hospital, Frankfurt, Germany; 2 Molecular Signal Transduction Laboratories, Institute for Microscopic Anatomy and Neurobiology, Focus Program Translational Neuroscience (FTN), Rhine Main Neuroscience Network (rmn2), University Medical Center of the Johannes Gutenberg University, Mainz, Germany; 3 Functional Proteomics, SFB815 Core Unit, Goethe-University, Frankfurt, Germany

**Keywords:** Nerve injury, Motor neuron, Progranulin, Locomotion, Notch, Proteomics

## Abstract

**Background:**

Peripheral nerve injury is a frequent cause of lasting motor deficits and chronic pain. Although peripheral nerves are capable of regrowth they often fail to re-innervate target tissues.

**Results:**

Using newly generated transgenic mice with inducible neuronal progranulin overexpression we show that progranulin accelerates axonal regrowth, restoration of neuromuscular synapses and recovery of sensory and motor functions after injury of the sciatic nerve. Oppositely, progranulin deficient mice have long-lasting deficits in motor function tests after nerve injury due to enhanced losses of motor neurons and stronger microglia activation in the ventral horn of the spinal cord. Deep proteome and gene ontology (GO) enrichment analysis revealed that the proteins upregulated in progranulin overexpressing mice were involved in ‘regulation of transcription’ and ‘response to insulin’ (GO terms). Transcription factor prediction pointed to activation of Notch signaling and indeed, co-immunoprecipitation studies revealed that progranulin bound to the extracellular domain of Notch receptors, and this was functionally associated with higher expression of Notch target genes in the dorsal root ganglia of transgenic mice with neuronal progranulin overexpression. Functionally, these transgenic mice recovered normal gait and running, which was not achieved by controls and was stronger impaired in progranulin deficient mice.

**Conclusion:**

We infer that progranulin activates Notch signaling pathways, enhancing thereby the regenerative capacity of partially injured neurons, which leads to improved motor function recovery.

**Graphical abstract:**

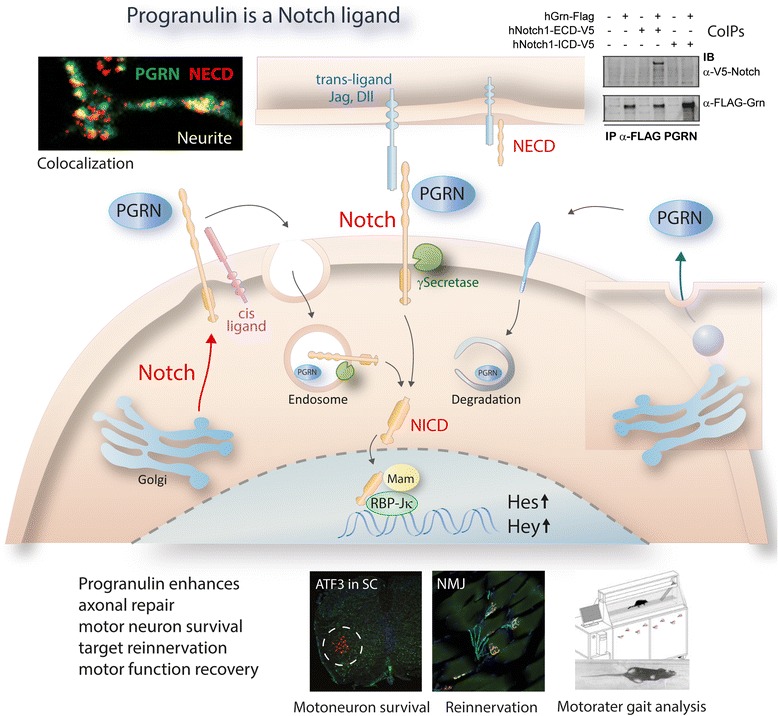

**Electronic supplementary material:**

The online version of this article (doi:10.1186/s13024-016-0132-1) contains supplementary material, which is available to authorized users.

## Background

Peripheral nerve injury or inflammation is a major cause of persistent chronic, pathological pain and muscular dystrophy, spasms and locomotor disability. The mechanisms resulting in neuropathic pain and failure to rebuild neuromuscular synapses are not fully understood. It is believed that the velocity of successful re-innervation determines the risk of developing chronic neuropathic pain, which is often associated with maintenance of muscle weaknesses or cramping [1–4]. Peripheral nerves have an inherent capability to regrow and re-innervate target tissues, but to find the right path, they need intact Schwann cells and guiding cues of the target tissue, intact glia and neighboring neurons. The interplay of growth cone extension and collapse and the distance between the proximal nerve stump and its target region are critical for re-innervation but even after rebuilding of sensory nerve endings and neuromuscular synapses chronic pain and muscle weaknesses may persist because of lasting mal-adaptations including neuronal death, glia activation and synapse remodeling [5–7].

Endogenous mechanisms exist, which counteract these maladaptive changes, thus helping to regain stability and function in the sensory and motor axis. One of these protective endogenous mechanisms is promoted by progranulin [8]. Particularly, motor dysfunctions after nerve injury are irreversible in progranulin deficient mice [8], and progranulin deficient primary neurons badly attach to laminin and build weak dendritic trees in culture [8, 9], whereas recombinant progranulin is able to maintain survival in nerve growth factor depleted cultures [8]. Although our previous study provided some evidence for a role of progranulin in motor function recovery after nerve injury [8], its mechanisms in terms of nerve regeneration and re-innervation are still elusive. Progranulin is a secretory cysteine-rich protein consisting in seven and a half granulins [10, 11], which is produced by neurons and immune cells of myeloid lineages including microglia [12] and likely acts as autocrine and paracrine growth factor. It is highly expressed in motor neurons [9] and in glia surrounding injured motor neurons [8]. Secreted progranulin may be taken up with help of sortilin [13], or may bind to and activate a neurotrophic receptor, but so far a receptor transducing pro-growth or pro-survival signals of progranulin has not been found. Some data suggest that anti-inflammatory effects of peripheral progranulin are mediated by inhibition of tumor necrosis factor alpha (TNFα) receptor signaling [14]. However, anti-TNFα effects do not account for the neuroinflammatory phenotype of progranulin knockout mice [15] and are likely irrelevant for progranulin’s effects in the context of frontotemporal dementia, which is caused by loss-of-functions mutations in the progranulin gene. The growth promoting effects of recombinant progranulin were also independent of sortilin [16, 17], suggesting that sortilin only accepts endogenous progranulin or requires some help or pre-processing.

The structure of the granulins consists of a parallel stack of β-hairpins and a folding subdomain, which is shared by epidermal growth factor (EGF)-like protein modules [11, 18]. The three-dimensional structures of granulins and EGF are partially super-imposable [19], but granulins likely do not bind directly to EGF receptors [20]. The amino acid sequence dissimilarity of granulins and EGF rather suggests a parallel evolution towards solutions of common biological problems. However, both systems may interact and affinity capture mass spectrometry has indeed retrieved progranulin as member of the EGFR interactome [21]. Further high-throughput affinity-purification MS identified interactions of progranulin with Notch ligands [22], suggesting affinity to EGF like domains and pointing to Notch as putative receptor candidate. Notch receptors would fit well as receptors for progranulin’s functions as neurotrophin [23, 24], silencer of activated microglia [25] and immunmodulator, and this would also agree with its pro-angiogenic and tumor-promoting properties [26].

In the present study we used newly generated tamoxifen-inducible neuronal progranulin transgenic mice (SLICK-Grn-OE; SLICK [27]) to assess if neuron-specific overexpression of progranulin improved axonal regeneration and neuromuscular re-innervation. Mechanistically, we addressed the Notch hypothesis using (i) an unbiased proteomic approach (ii) co-immunoprecipitation studies and (iii) transcriptional analyses of Notch target genes and finally we used a battery of behavioral analyses to assess the functional relevance in vivo.

## Results

### Progranulin overexpressing mice

**Fig. 1:**
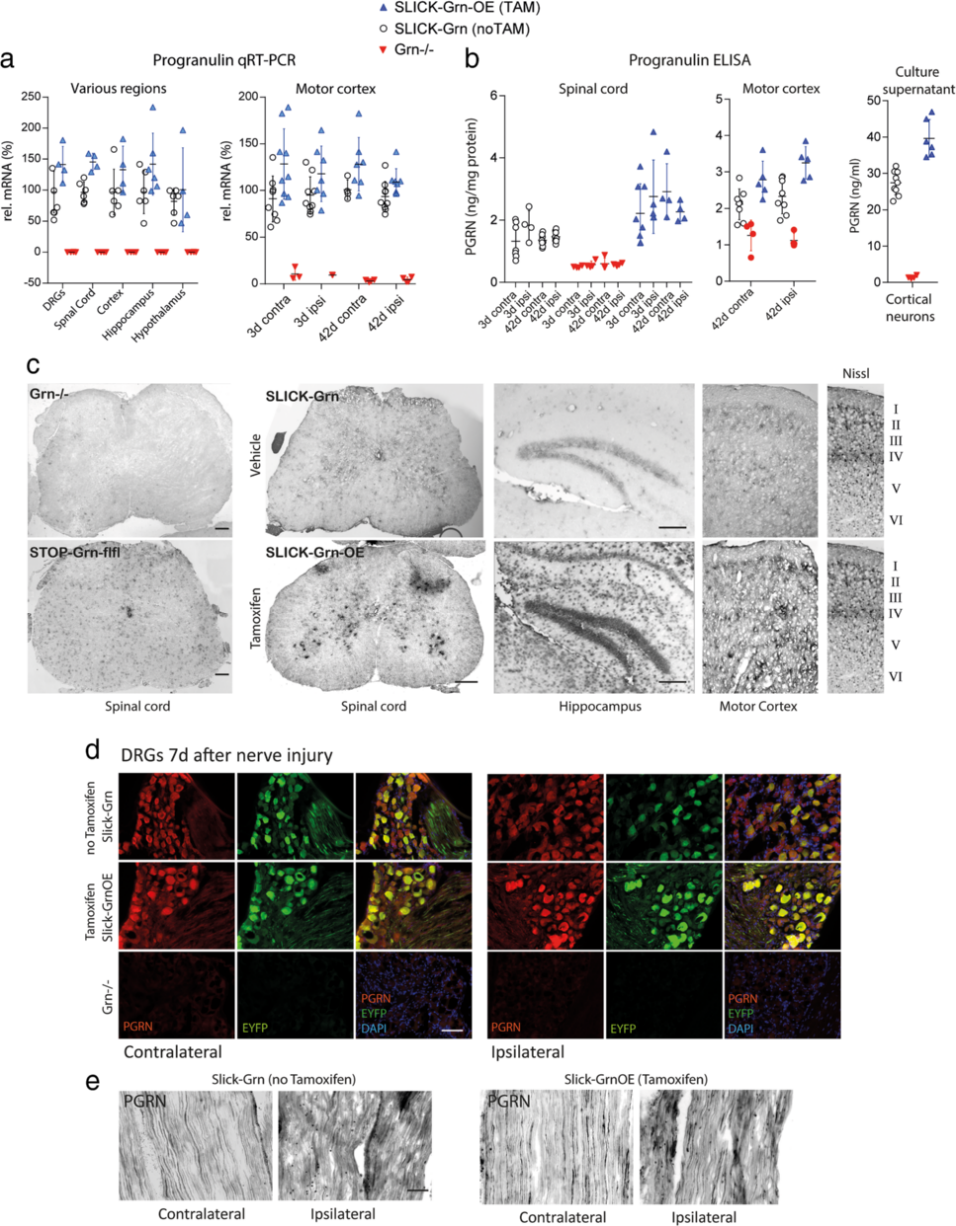
Characterization of tamoxifen inducible neuronal progranulin overexpressing mice. SLICK-Grn-OE mice were generated by mating homozygous STOP-Grn^flfl^ mice (progranulin cDNA with leading STOP sequence into Rosa26 locus) with SLICK-H Cre mice, resulting in overexpression of progranulin in the majority of central and peripheral neurons after tamoxifen treatment. **a** Quantitative RT-PCR of progranulin mRNA in dorsal root ganglia (DRGs), spinal cord, brain regions and motor cortex, the latter after sciatic nerve injury in SLICK-Grn mice (without tamoxifen, noTAM, = control) and SLICK-Grn-OE mice (with tamoxifen, TAM). Progranulin knockout (Grn^−/−^) mice show the primer specificity. The contralateral motor cortex is the cortical representation of the injured nerve. Each symbol is a mouse, the line is the mean and whiskers show the standard deviation (SD). **b** Enzyme immunoassay of progranulin in spinal cord, brain and primary culture supernatants in SLICK-Grn (without tamoxifen) and SLICK-Grn-OE (with tamoxifen) mice. Grn^−/−^ mice show the antibody specificity. The figure shows scatter plots of individual mice with mean ± SD. Tamoxifen treatment significantly increased Grn mRNA and protein expression throughout regions (rm-ANOVAs for genotype *P* < 0.05) without significant differences between the ipsilateral and contralateral sides. **c** In situ hybridization of progranulin mRNA in the spinal cord, hippocampus and motor cortex of Grn^−/−^ mice, STOP-Grn^flfl^ mice, SLICK-Grn (without tamoxifen) and SLICK-Grn-OE (with tamoxifen) mice. **d** Immunofluorescence analysis of EYFP and progranulin in SLICK-Grn mice (without tamoxifen) and SLICK-Grn-OE mice (with tamoxifen) in the DRGs. The double-headed Thy1 promotor drives constitutive EYFP expression and inducible CreERT-dependent expression of Cre recombinase that cuts out the STOP codon in front of progranulin cDNA. Hence, progranulin expression increases after tamoxifen, EYFP remains constant. In situ and immunofluorescence images are representative results of 3–4 mice per group. Scale bars: 50 μm. **e** Immunofluorescent analysis of progranulin in injured (ipsilateral) and uninjured (contralateral) sciatic nerves in SLICK-Grn mice (without tamoxifen) and SLICK-Grn-OE mice (with tamoxifen). Full time courses are shown in Additional file 1: Figure S2. Scale bars: 50 μm

We generated mice with tamoxifen-inducible overexpression of progranulin in the majority of central and peripheral neurons (SLICK-Grn-OE) (Fig. 1) by mating homozygous floxed mice with SLICK-H Cre mice [27]. Recombination was confirmed by PCR-based genotyping (not shown). SLICK-Cre mice express Cre-recombinase under control of a double headed Thy1 promoter, one head driving CreERT and the other EYFP expression. Hence, Cre positive neurons that are capable to overexpress proganulin after tamoxifen treatment are green. Quantitative RT-PCR of dorsal root ganglia (DRGs), spinal cord, and brain regions of adult mice confirmed the expected increase of progranulin mRNA by about 50–100 % after tamoxifen treatment (Fig. 1a). Tamoxifen had no effect on progranulin expression in C57BL6 mice (Additional file 1: Figure S1). CreERT-mediated progranulin upregulation after tamoxifen was also shown using ELISA or immunofluorescence analyses (Fig. 1b, d, e and Additional file 1: Figure S2). To further rule out effects of tamoxifen we show a similar Cre-mediated progranulin upregulation in Nestin-Grn-OE mice versus STOP-Grn^flfl^ mice (Additional file 1: Figure S3a-c). Nestin-Cre mice express Cre recombinase in neural stem cells, hence nestin-mediated recombination commences around emryogenic day E11 and does not require tamoxifen.

Nerve injury did not significantly alter progranulin expression in the spinal cord or motor cortex (Fig. 1b). The tamoxifen inducible SLICK-Cre mediated overexpression was further confirmed by in situ hybridization (Fig. 1c) and immunofluorescence studies in dorsal root ganglia (DRGs) (Fig. 1d) and sciatic nerves (Fig. 1e). In progranulin knockout mice neither mRNA nor protein were detected, confirming the specificity of the primers, in situ probes and the antibody. In the sciatic nerve, progranulin was expressed in some fibers suggesting its transport along axons (Fig. 1e). After nerve injury, progranulin immunoreactive immune cells invaded the crushed nerve (full time course Additional file 1: Figure S2), whereas its expression in axonal fibers within the lesion rather decreased.

### Progranulin promotes motor neuron survival and regrowth after sciatic nerve injury

**Fig. 2:**
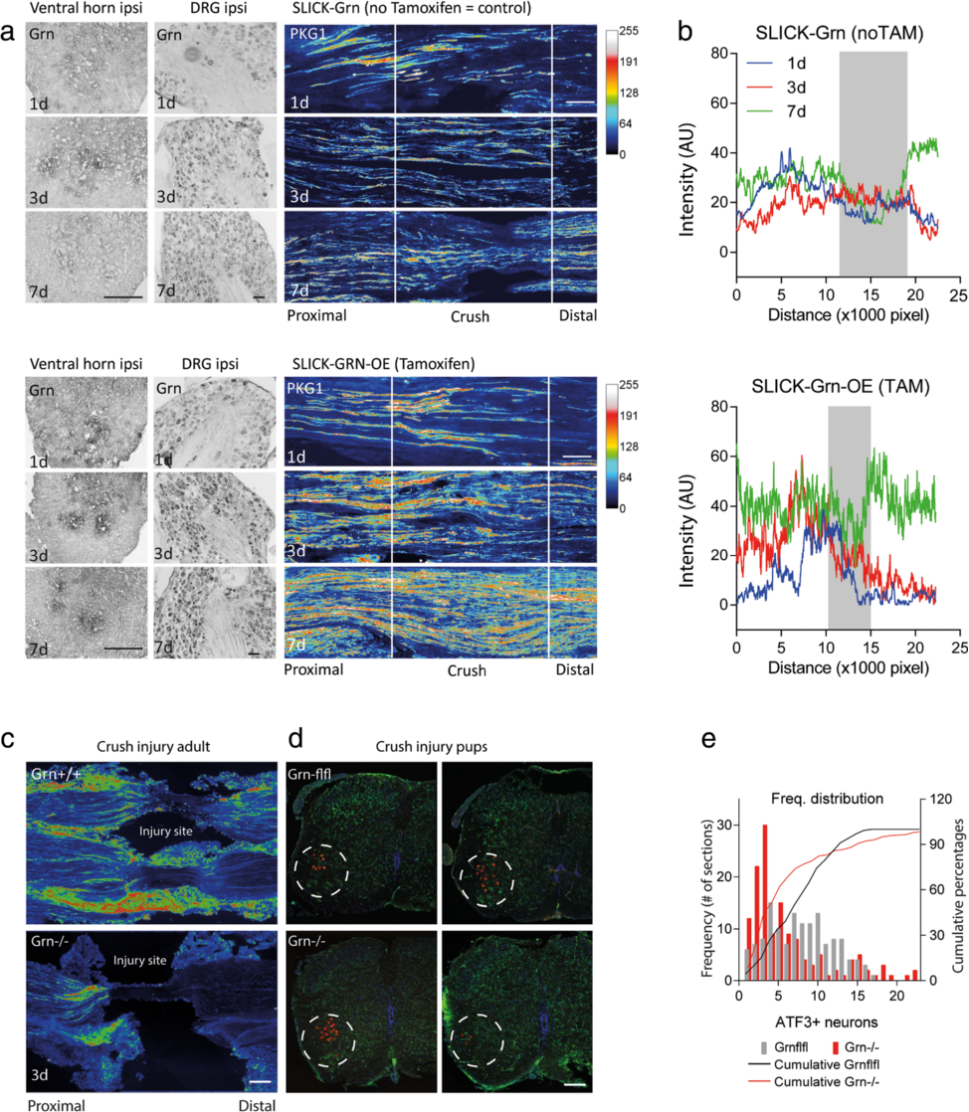
Immunofluorescent analysis of axonal regrowth and motor neuron survival after injury of the sciatic nerve. **a** Cyclic GMP dependent protein kinase (PKG1) immunofluorescence 1, 3 and 7 days after crush injury of the sciatic nerve in SLICK-Grn mice (without tamoxifen) and SLICK-Grn-OE mice (with tamoxifen). PKG1 is a marker of axonal transport and regrowth. The images are rainbow pseudo-color images of fluorescent intensities. The left panels are in situ hybridizations showing progranulin mRNA expression of the respective DRGs and ventral horns of the spinal cord. The figures show representative results of *n* = 3 per time point. Scale bars 100 μm ventral horn, 50 μm DRGs and 200 μm nerves. **b** 2D intensity profile plots showing the quantitative analysis of PKG1-immunofluorescence in the sciatic nerves (exemplary result of *n* = 3 per time point). The grey area shows the remaining gap at 7days. PKG1 initially accumulates in front of the lesion and then approaches towards the distal end with the outgrowing fibers, when the axonal transport is resumed. **c** PKG1 immunofluorescent intensity (rainbow pseudo-color) of the lesion site of the sciatic nerve in adult progranulin deficient and control mice. There was no regrowth and a long gap in Grn^−/−^ mice. Scale bar 100 μm. Images are examples of 3–4 mice per group. **d** ATF3 immunofluorescence of injured motor neurons in the ventral horn of the spinal cord of Grn^−/−^ and Grn^+/+^ mice after sciatic nerve injury in pups showing each 2 representative images. ATF3 is a marker of axonal injury, neurons are stressed but alive. Grn^−/−^ either had a high number of ATF3 positive neurons or had lost these neurons. The bimodal distribution is reflected in the frequency distribution plots in e. **e** Quantitative analysis of ATF3 positive neurons showing the frequency distribution of ATF3 positive neurons per section. 142 sections of 6 mice were analyzed per group. The number of ATF3+ neurons per section followed a normal distribution in Grn^+/+^ mice, but was left-skewed in Grn^−/−^ mice showing that ATF3+ neurons had died. In Grn^−/−^ mice, there was a second peak representing sections with high numbers of ATF3+ neurons. The cumulative frequency distributions were statistically compared using the two sample Kolmogorov-Smirnov test. The null hypothesis that both distributions were equal was rejected

**Fig. 3:**
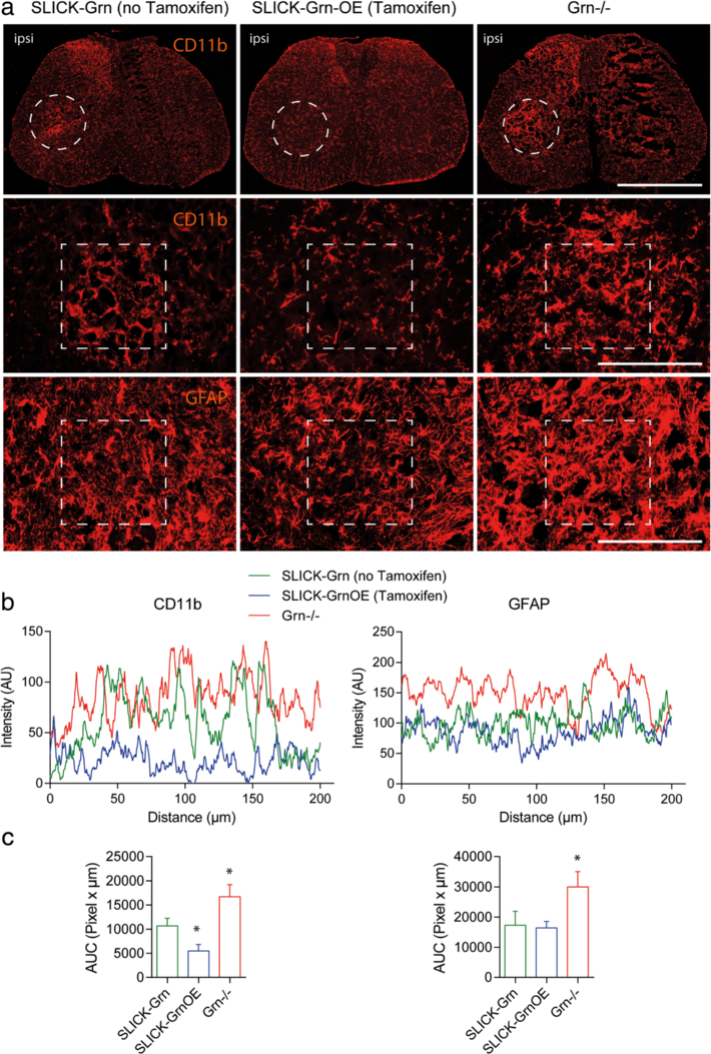
Immunofluorescent analysis of glia activation in the spinal cord after crush injury of the sciatic nerve. **a** CD11b (microglia) and GFAP (astrocytes) in SLICK-Grn mice (without tamoxifen), SLICK-Grn-OE mice (with tamoxifen) and Grn^−/−^ mice. The circled area in the upper panel showing the glia activation around the injured motor neurons was used for magnification. Scale bars 200 μm. **b** 2D intensity profile plots of the rectangles shown in the middle and bottom panels in a. The AUCs of the profile plots differed significantly between groups **c** AUCs of the intensity profile plots (*one-way ANOVA, *P* < 0.05, posthoc Dunnett versus SLICK-Grn (without tamoxifen) control mice, *n* = 3)

**Fig. 4:**
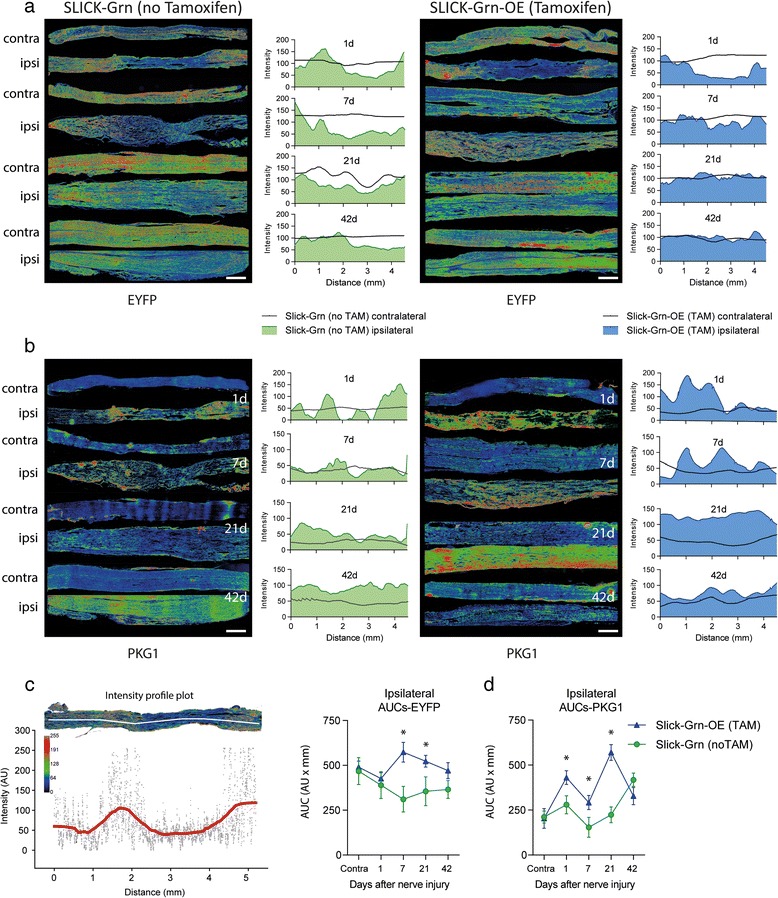
Immunofluorescent analysis of long-distance axonal regrowth after crush injury of the sciatic nerve. **a** Time course of rainbow pseudo-color images of EYFP immunofluorescent intensities along the injured sciatic nerve in SLICK-Grn mice (without tamoxifen) and SLICK-Grn-OE mice (with tamoxifen). Tiled images were used for reconstruction of the nerves. The 2D line intensity plots show EYFP intensity of the ipsilateral (filled area) and the contralateral nerve (line), which was used as control (procedure of quantification in c). Scale bars 500 μm. **b** PKG1 immunofluorescent intensities and quantification in analogy to a. **c** Illustration of the procedure of intensity quantification along the nerve. Line intensity profile plots through the middle of the nerve were obtained in ImageJ after background subtraction and automatic setting of the intensity threshold, then exported and smoothed employing the Loess function. **d** Time courses of the areas under the smoothed intensity profile plots (AUCs), calculated according to the linear trapezoidal rule (means ± SD; * *P* < 0.05, rm-ANOVA with subsequent *t*-tests employing a Bonferroni correction of alpha)

**Fig. 5:**
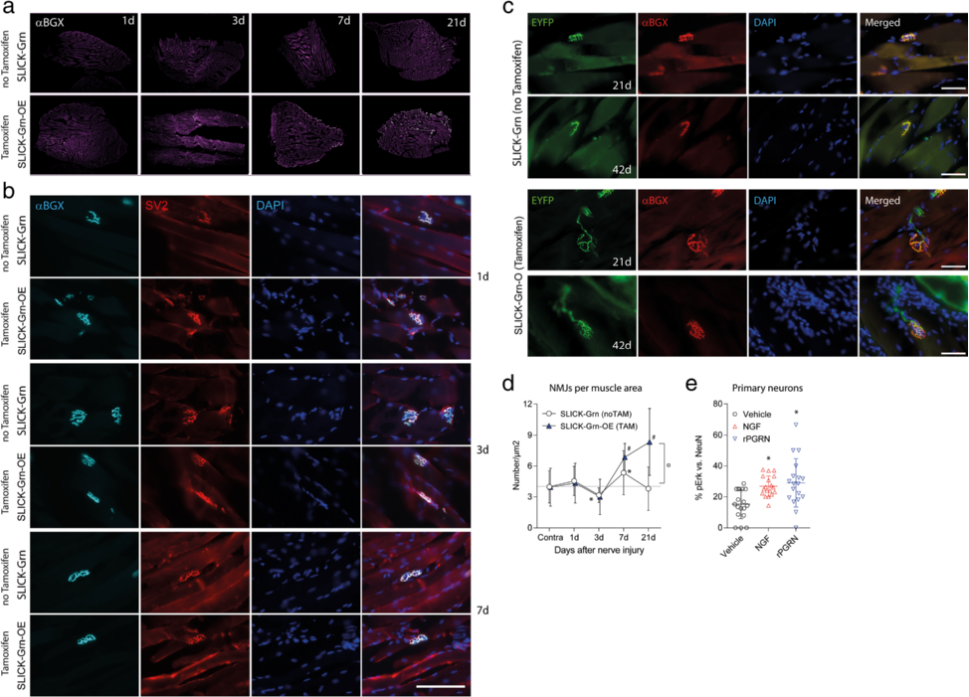
Morphology and time course of neuromuscular synapses after crush injury of the sciatic nerve. **a** Overview of alpha-bungarotoxin-Alexa Fluor 594 (αBGX) stained neuromuscular junctions (light dots) in muscle sections of the ipsilateral gastrocnemius muscles after crush injury of the sciatic nerve of SLICK-Grn mice (without tamoxifen) and SLICK-Grn-OE mice (with tamoxifen). **b** Examples of the time course of neuromuscular junctions. The presynaptic site was identified by immunostaining of the synaptic vesicle protein, SV2; the postsynaptic site by labeling of nicotinic acetylcholine receptors with αBGX. Scale bars 200 μm. **c** Examples of neuromuscular junctions 21 and 42days after nerve injury. NMJs were identified via EYFP and αBGX to detect the nicotinic acetylcholine receptor. The EYFP signal arises from the regrowing axon and shows the presynaptic site. The gross morphology of the neuromuscular junctions was similar in control and tamoxifen treated progranulin overexpressing mice, but the number of intact NMJs was higher in the latter. Scale bars 50 μm. **d** Counts of αBGX-positive neuromuscular junctions in gastrocnemius muscles after crush injury of the sciatic nerve per muscle area (means ± SD). The contralateral sides served as controls (all time points summarized). The asterisks (*) and crosses (#) mark time dependent significant changes in the counts of ipsilateral NMJs per square μm of the muscle versus contralateral. The circle shows a significant difference between groups. Thirty to forty sections were analyzed per group, per time point and represent results of 5–6 mice per group and time point. *P* < 0.05 for all tests. **e** Erk phosphorylation (pErk immunofluorescence) in primary DRG neurons stimulated with 50 nM nerve growth factor, NGF or 10 ng/ml recombinant human progranulin (rPGRN) in the absence of NGF. The number of pErk positive and NeuN positive neurons were counted per frame, 2–3 frames per culture (Axiovision autmess software). Data are means ± SD percentages of pErk + neurons. Asterisks show significant increases of pErk positive neurons versus unstimulated neurons (one-way ANOVA, *P* < 0.05, posthoc Dunnett versus unstimulated). rPGRN and NGF had similar effects

We used the sciatic nerve crush injury model to assess effects of progranulin in terms of axonal transport and regrowth, motor neuron survival, glia activation, re-innervation and functional recovery (Figs. 2, 3, 4 and 5). After crush injury, motor neurons of control mice lost some progranulin expression, which did not occur in SLICK-Grn-OE mice (Fig. 2a, left panel). Progranulin expression in the DRGs mildly increased in both groups, but was stronger in tamoxifen treated SLICK-Grn-OE mice than in controls (Fig. 2a, middle panel). Tamoxifen-induced overexpression of progranulin was associated with enhanced and accelerated outgrowth of fibers through the crush site, as assessed by cGMP dependent protein kinase 1 (PKG1) immunofluorescence, which is a marker for axonal transport and regrowth (Fig. 2a right panel pseudocolors, b intensity plot). The intensity of PKG1 proximal of the lesion is a measure of its accumulation due to the block of axonal transport, its intensity within the lesion shows the regrowth leading to a reduction of the gap size over time (grey shaded area in Fig. 2b). A similarly accelerated time course of fiber degradation, debris removal and start of regrowth was observed in Nestin-Grn-OE versus STOP-Grn^flfl^ mice (Additional file 1: Figure S3). The process starts with a hypodense areal, then fragmented fiber morphology, fiber swelling, followed by increased fiber intensity at the proximal border reaching into the lesion. The nerves of the Nestin-Grn-OE mice were always a step ahead of the controls. This was also reflected in the NF200 and PKG1 fiber intensity at the proximal border and inside of the lesion and in the RotaRod tests showing the improvement of the motor function recovery.

In progranulin deficient mice, the site of the nerve injury was without signs of regrowth at 3 days, there was a long gap and strong proximal accumulation of PKG1 (Fig. 2c) suggesting a reduction of the regenerative capacity and likely enhanced number of dying neurons. To address the latter question we counted ATF3 positive neurons in the ventral horn after crush injury in pups, which allows for a quantitative assessment of motor neuron death [28] (Fig. 2d, e). ATF3 is a marker of neurons with axonal injury [29], which are stressed but may recover. Progranulin deficient mice clustered into two groups: either they had high numbers of ATF3 positive motor neurons or they had lost these neurons. In contrast, AFT3 positive neurons were normally distributed in the control animals (Fig. 2e). Skewness and curtosis of the frequency distribution curves differed significantly between groups (Skewness: Grn^+/+^ 0.197 ± 0.208; Grn^−/−^ 1.621 ± 0.203; Curtosis: Grn^+/+^ −0.822 ± 0.413; Grn^−/−^ 2.120 ± 0.404).

### Progranulin overexpression reduces neuroinflammation in the ventral horn

Stress and death of neurons after sciatic nerve injury is contributed by surrounding neuroinflammation in the dorsal and ventral horns (Fig. 3a, quantification b, c). The extent of the glia activation and proliferation agrees well with readouts of sensory and motor dysfunctions and therefore is a good indicator of the extent of the damage. Microglia and astroglia activation after nerve injury was intensified in progranulin deficient mice and was attenuated in SLICK-Grn-OE mice (Fig. 3a, exemplary intensity plots in b and AUCs in c). The result agrees with a pro-neuroinflammatory state in progranulin deficient mice in models of brain ischemia or injury [30, 31].

### Progranulin overexpression improves axonal regeneration and recovery of neuromuscular junctions

Enhanced local fiber regrowth does not necessarily lead to a better re-innervation, but may also lead to aberrant sprouting in case of incorrect or missing guidance. To distinguish the first from the latter we analyzed fiber regrowth via EYFP and PKG1 immunostainings using reconstructions of long distances of the sciatic nerve and quantification via line intensity plots (Fig. 4a, b; procedure of quantification c, AUCs d). Both EYFP and PKG1 analyses revealed an accelerated regrowth in tamoxifen treated progranulin overexpressing SLICK-Grn-OE mice. They were about 3 weeks ahead of the control mice. At 6 weeks, the ipsilateral side had fully recovered the normal EYFP and PKG1 intensity and distribution of the contralateral side. This normalization was not reached in the SLICK-Grn control animals. Six weeks after the injury, there was still a gap for EYFP and intensified staining for PKG1 in the control mice showing that regeneration was still in progress.

Similar experiments up to 7days after nerve injury were done in Nestin-Grn-OE versus STOP-Grn^flfl^ mice (Additional file 1: Figure S3d-f) using immunofluorescence analyses of NF200, PKG1 and peripherin. NF200 is a marker for neurons with myelinated fibers including motor neurons, whereas PKG1 and peripherin predominantly label small to medium sized DRG neurons with unmyelinated or myelinated fibers and motor neurons. Again, progranulin overexpressing mice recovered fiber density faster, best seen with NF200, suggesting faster regrowth.

To assess if the enhanced fiber regrowth resulted in faster re-innervation of the target we analyzed neuromuscular junctions (NMJs) in the gastrocnemius muscles after crush injury of the sciatic nerve (Fig. 5a overview). The contralateral side served as control (not shown). Functional NMJs were identified by co-staining of the presynaptic synaptic vesicle protein SV2 and the postsynaptic nicotinergic acetylcholine receptors with alpha bungarotoxin (αBGX) (Fig. 5b). The NMJs were also EYFP positive showing the Thy1-driven EYFP expression in all motor neurons, provided by the SLICK-H-Cre (Fig. 5c). The gross morphology of αBGX/SV2 positive NMJs or of αBGX/EYFP NMJs was similar in SLICK-Grn-OE and control mice (Fig. 5b, c) but after nerve injury, their numbers differed. In control mice, αBGX positive NMJs were reduced 3 days after injury (Fig. 5d), followed by an increase at 7 days and return to the contralateral level at 21 day (ANOVA with posthoc Dunnett versus contralateral F = 10.247, df = 4, *P* < 0.0001, posthoc in the figure). Tamoxifen treated SLICK-Grn-OE mice did not show the initial drop and had a strong overshooting increase at day 7 and 21 (ANOVA with post hoc Dunnett versus contralateral F = 36.967, df = 4, *P* < 0.0001, posthoc in the figure) resulting in a significantly higher number of NMJs at 21 day compared with the control group (ANOVA F = 25.15814, df = 9, *P* < 0.0001, posthoc *t*-test Bonferroni adjusted for 21day *P* < 0.0001). Hence, progranulin was neurotrophic for injured motor neurons, directly or indirectly. In support, treatment of primary neurons with recombinant progranulin elicited phosphorylation of Erk (Fig. 5e), a downstream signal, on which growth factor stimulation converges.

### Progranulin and notch signaling

**Fig. 6:**
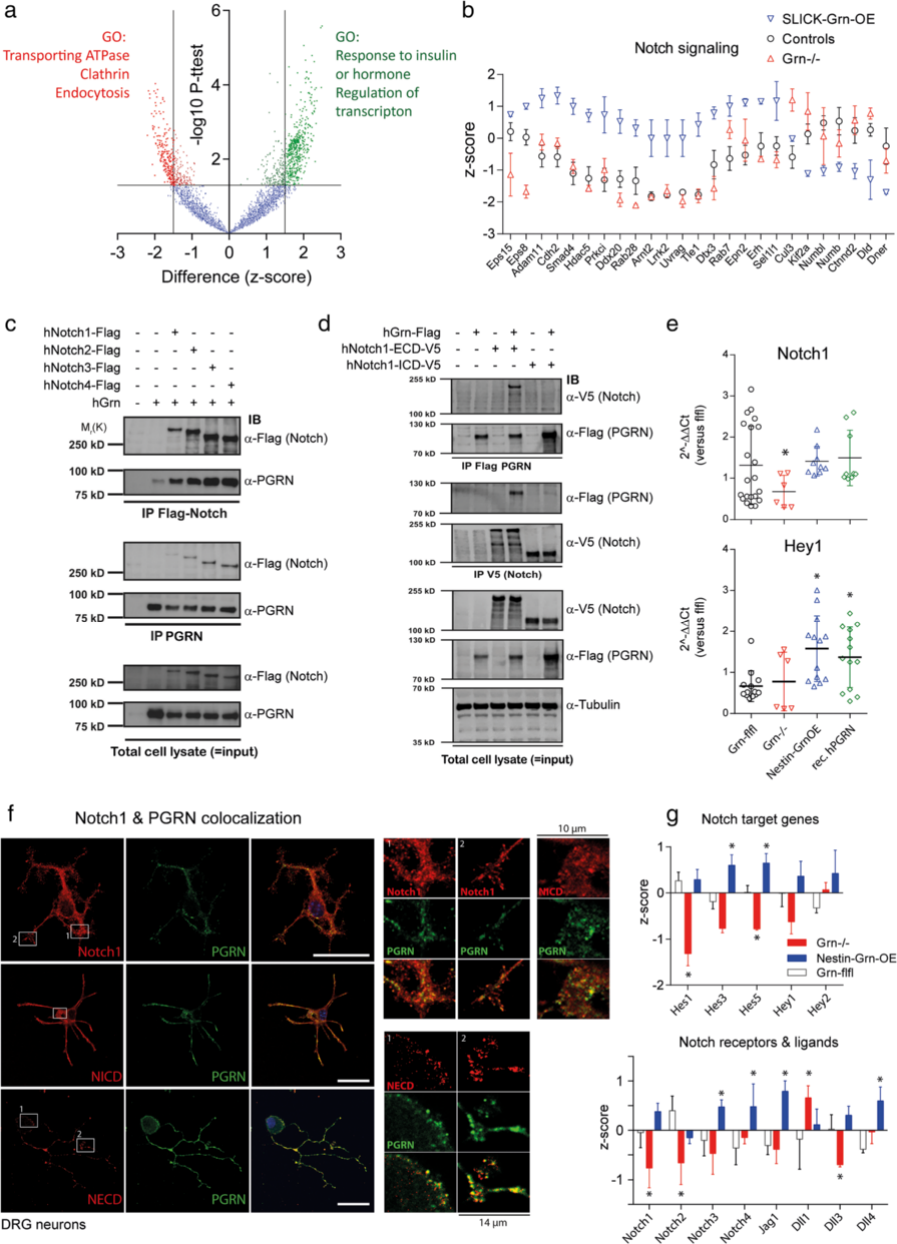
Proteome analysis and assessment of progranulin—Notch interactions. **a** Volcano plot showing differentially expressed proteins in the prefrontal cortex of SLICK-Grn-OE (*n* = 3) versus control mice (*n* = 6). LN-transformed LFQ (label free quantification) values were z-transformed and then submitted to 2-tailed independent *t*-tests, using FDR adjustments. The –log *P* values were plotted on the Y-axis versus the z-score difference on the x-axis. Upregulated proteins are shown in the upper right corner, downregulated proteins in the upper left corner. Significantly up- or downregulated proteins (*P* < 0.05; = − logP > 1.3) are highlighted in green or red color, respectively and were subsequently submitted to Gene Ontology (GO) enrichment analysis (genetrail); full results in Additional file 2: Table S1. **b** Scatter plots (mean and SD of the z-scores) of significantly regulated proteins, which are involved in Notch signaling or traffic. The list of regulated proteins was searched for proteins involved in Notch signaling or traffic by comparing them with Notch networks (biogrid, genemania) and with proteins that have GO annotations of “Notch signaling” (*n* = 3 per group, *n* = 6 for controls). **c** Co-immunoprecipitation analysis of interactions between Notch receptors and progranulin. HEK293 cells were transiently co-transfected with Flag-tagged human Notch receptors and full-length non-tagged human progranulin (hGrn). Pull-down was performed with anti-Flag (for Notch receptors) or anti-progranulin (N19 antibody) followed by immunoblot analysis with anti-Flag and anti-progranulin. Input blots with total protein lysates were done with 1/10^th^ of the protein subjected to IP. Input blots of total protein lysates are shown in the bottom. **d** Co-immunoprecipitation analysis of interactions between the extracellular or intracellular domains (NECD and NICD) of human Notch1 with human progranulin. HEK293 cells were transiently co-transfected with hNotch1-ECD-V5 or hNotch1-ICD-V5 and Flag-tagged full-length human progranulin (hGrn-Flag, all tags were C-terminal). IPs were performed with anti-Flag and anti-V5 followed by immunoblot analysis with anti-V5 (for the NECD, NICD) and anti-Flag (for progranulin, PGRN). Input blots were done with 1/10^th^ of the protein and beta tubulin served as loading control for input blots. **e** Scatter plots of Notch1 and Hey1 mRNA (QRT-PCR) in primary embryonic neuron cultures of Nestin-Grn-OE, Grn^−/−^ and control mice, the latter without/with stimulation with recombinant human progranulin (rPGRN). Each symbol represents a culture, the line is the mean, the whiskers show the SD. Rps13 was used as housekeeping gene for calculation of ΔCt values. Asterisks show significant differences versus unstimulated control cultures. * *P* < 0.05, one-way ANOVA, posthoc *t*-tests according to Dunnett versus control. **f** Immunofluorescence wide-field laser scanning microscopic analysis of the co-expression of progranulin with Notch1, the NICD and the NECD of Notch1 in primary neurons of the dorsal root ganglia of adult STOP-Grn^flfl^ mice. Scale bar 25 μm. The right panels show zoom-in images of the white rectangular regions (width 10 μm for Notch1 and NICD and 14 μm for NECD). Quantitative colocalization analysis revealed partial overlap (Additional file 2: Table S4). **g** Bar charts showing Z-cores (means + SD) of QRT-PCR mRNA levels of Notch target genes, Notch receptors and Notch ligands after nerve injury in DRGs. Results of ipsilateral and contralateral DRGs were summarized. Data are of *n* = 3 samples per genotype. Each sample consisted of the ipsilateral or contralateral L4 and L5 DRGs of each 3 mice. Hence, the analysis is based on tissue of 18 mice with 36 ipsilateral and 36 contralateral DRGs. Each sample was analyzed in 3 replicate RT-PCR runs for each gene. Two housekeeping genes, RNA Pol-III and Rps13, were used for calculation of ΔCt values. The ΔCt data of the respective genes were Z-transformed for each gene separately. Z-transformed data were submitted to 2-way ANOVA for “gene” by “genotype” and groups differed significantly with *P* = 0.0005. Subsequently, one-way ANOVAs were performed for each gene individually with subsequent post-hoc analyses according to Dunnett versus STOP-Grn^flfl^ control mice. Asterisks show statistically significant results, * *P* < 0.05

To assess potential underlying molecular mechanisms, we performed a proteomic screen and gene ontology (GO) enrichment analysis to identify proteins, pathways and functions that are influenced by progranulin. The mass spectrometry proteomics raw data have been deposited to the ProteomeXchange Consortium via the PRIDE partner repository with the dataset identifier PXD004087. In the prefrontal cortex of SLICK-Grn-OE mice, 474 out of 5022 proteins were significantly upregulated and 210 proteins were downregulated (Fig. 6a), including a number of proteins involved in Notch signaling or traffic (Fig. 6b). Controls (Grn^+/+^ and SLICK-Grn without tamoxifen) were combined to increase power because there were only minor differences. The GO analysis of proteins, which were upregulated in SLICK-Grn-OE mice, revealed an enrichment of the terms “regulation of transcription” and “response to insulin” and similar terms (Additional file 2: Table S1). Downregulated proteins showed an enrichment of the terms “(transporting) ATPase”, “endocytosis” and “clathrin complex”, pointing to changes of vesicle transport. Because of the enrichment of “transcription” we used a transcription factor (TF) prediction tool (Tfacts (http://www.tfacts.org) [32]) to assess, which TFs were likely active. The search predicted Rbp-jkappa to be active. Rbpj is essential for transcriptional functions of Notch. In addition, the search predicted that basic helix-loop-helix (bHLH) transcription factors, which are downstream targets of Notch were active including Hes1 and Myc. We therefore searched the set of regulated proteins for proteins that have GO annotations for “Notch receptor signaling” (AmiGO; http://amigo.geneontology.org) or are involved in Notch endocytosis (Fig. 6b). The pattern of regulated Notch associated proteins supported the hypothesis of a progranulin-Notch crosstalk.

### Immunoprecipitation and colocalization of progranulin and notch and notch target genes

We next assessed whether this interaction may be direct by binding of progranulin to Notch receptors. Immunoprecipitation (IP) of human Notch1, 2, 3 and 4 using anti-Flag-Notch retrieved human progranulin (Fig. 6c). Vice versa, IP with anti-progranulin (PGRN) retrieved all of the four Notch receptors (Fig. 6c). To assess the site of binding we performed Co-IPs with the extracellular (ECD) and intracellular domains (ICD) of human Notch1 tagged with V5 with human progranulin-Flag. PGRN-Flag was retrieved after IP with anti-V5-Notch1-ECD but not anti-V5-Notch1-ICD and inversely, anti-Flag-PGRN IP pulled down the NECD but not NICD (antibodies and vectors in Additional file 2: Tables S2, S3). Hence, progranulin bound to the extracellular domain of the Notch1 receptor. (Fig. 6d). The Co-IPs were repeated and confirmed with the respective mouse proteins (mNotch1 ECD-V5, mNotch ICD-V5, mGrn-Flag) (Additional file 1: Figure S4). A graphical illustration of the putative signalling is shown in the Graphical Abstract.

We further assessed a progranulin-Notch crosstalk in primary cortical neurons. Neurons of Grn^−/−^ mice showed reduced Notch1 mRNA whereas overexpression of progranulin or stimulation with recombinant human progranulin increased expression of the Notch target gene Hey1 (Fig. 6e), showing that progranulin was required to maintain Notch1 expression, and that increased levels of progranulin enhanced Notch-dependent gene transcription.

Double immunofluorescence analyses in primary DRG neurons of adult STOP-Grn^flf^ mice showed that progranulin partially co-localized with mature Notch1 at the cell surface and intracellularly in vesicular structures (Fig. 6f). Vesicular structures were also double positive for progranulin and the NICD, whereas NECD mostly colocalized with progranulin at the cell surface. The localization is in line with the idea that progranulin binds to the extracellular domain and is internalized together with Notch, which may then be processed in the endosome or lysosome [33, 34]. Results of a quantitative assessment of the colocalization using the JACoB plugin of FIJI ImageJ [35] are presented in Additional file 2: Table S4. The strongest colocalization occurred with the NECD.

To assess functional implications of the interaction we performed a transcriptional analysis of Notch receptors, ligands and Notch target genes in the DRGs after nerve injury (Fig. 6g). The analysis was based on *n* = 3 samples per genotype, consisting each in L4 plus L5 ipsilateral or contralateral DRGs of 3 mice. Nestin-Grn-OE mice were used in this experiment to avoid putative influences of tamoxifen. At the mRNA level we found high expression of Notch1 and 2, Jag1, Dll1, Hes1 and Hey1 and 2 in the DRGs whereas mRNA expression of Notch3 and 4 and Dll3 and 4 was low. Jagged2 was not detected. For further analyses ΔCt levels were transformed to Z-scores for each gene to allow for an assessment of genotype effects for genes with different expression levels. A heatmap of ipsilateral versus contralateral DRGs is shown in Additional file 1: Figure S4b. Seven days after nerve injury Dll1 mRNA was doubled ipsilateral versus contralateral in all genotypes and Hes1, 3, 5 tended to decrease. Two-way ANOVA for “genes” by “genotype” revealed significant differences between genotypes (F (5, 36) = 5.786; *P* = 0.0005). To increase power ipsilateral and contralateral DRGs were combined (Fig. 6g), and one-way ANOVAs with subsequent Dunnett post hoc analyses were performed for each gene individually. Nestin-Grn-OE mice showed higher mRNA levels of Notch receptors, Jag1 and target genes, whereas Grn^−/−^ mice mostly showed the opposite (post hoc results in Fig. 6g).

### Progranulin overexpression improves the recovery of motor functions after sciatic nerve injury

**Fig. 7:**
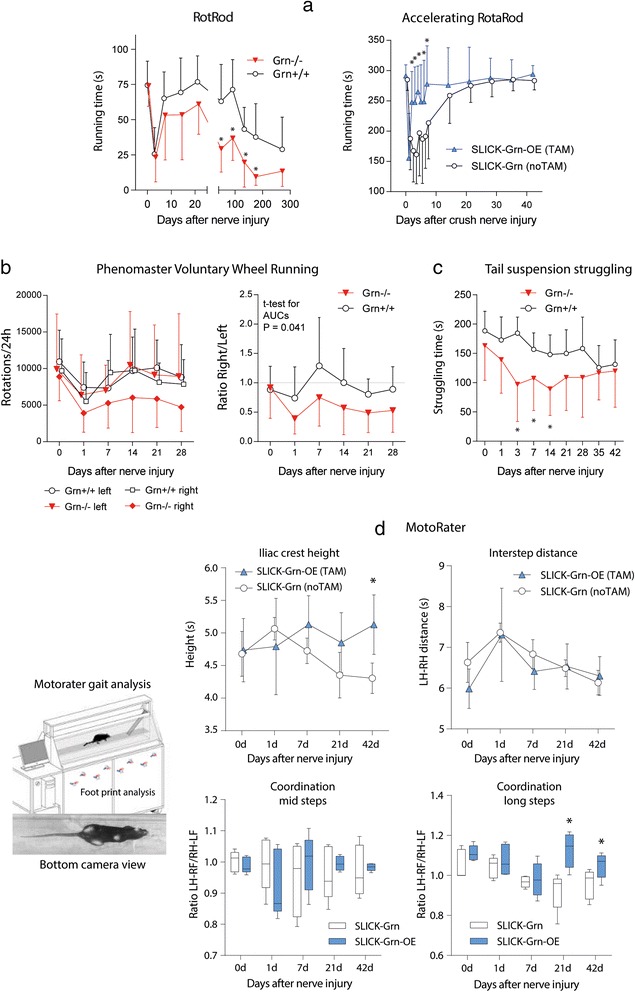
Motor function analysis before and after sciatic nerve injury in progranulin deficient, control and progranulin-overexpressing mice. Behavioral data are the means ± SD of *n* = 8–12 mice per group. **a** Time courses of the running times on a constant speed (Grn^−/−^) or accelerating RotaRod (SLICK-Grn-OE, with tamoxifen) before and after spared nerve injury or crush nerve injury of the sciatic nerve, respectively. Asterisks show significant differences between genotypes (rm-ANOVA and subsequent *t*-tests between genotypes, * *P* < 0.05). **b** Time course of voluntary wheel running before and after spared injury of the left sciatic nerve in Grn^−/−^ and Grn^+/+^ mice. Running times briefly dropped after nerve injury but rapidly recovered. After nerve injury, Grn^−/−^ avoided right turns and the right/left ratio significantly differed between genotypes (* *P* < 0.05; rm-ANOVA and subsequent 2-tailed *t*-tests between genotypes). **c** Time course of the struggling times in the tail suspension test (TST) before and after crush injury of the sciatic nerve. Struggling decreased over time in both groups. The decrease was stronger in Grn^−/−^ mice (* *P* < 0.05; rm-ANOVA, posthoc 2-sided *t*-tests versus control, Dunnett adjustment of alpha).**d** MotoRater analysis of locomotion in an over-ground walking task in SLICK-Grn-OE (with tamoxifen) and SLICK-Grn mice (without tamoxifen, control). The upper panel shows the time courses of the iliac crest height and hind paw inter-step distances before and after crush injury of the sciatic nerve (means ± SD). The lower box plots show the time course of coordination represented by the ratios of the diagonals of the ‘left hind to right front’ paw (LH-RF) and vice versa (LF-RH) for the steps of mid lengths (left) and the long steps (right). The line is the median, the box represents the interquartile range, the whiskers show 5 –95 % percentiles ( *P < 0.05; rm-ANOVA, subsequent 2-tailed *t*-tests between genotypes)

Finally, we assessed the functional consequences. Motor tests were employed to assess coordination, muscle strength and endurance, voluntary running and gait (Fig. 7). In the RotaRod test, progranulin deficient mice showed normal running times at baseline but after injury they had a stronger and longer lasting impairment compared with the controls, and Grn^−/−^ mice did not fully recover (Fig. 7a left; rm-ANOVA for ‘genotype’ F (1, 14) = 7.851; *P* = 0.014). Oppositely, tamoxifen-treated SLICK-Grn-OE mice recovered faster than their control littermates (Fig. 7a right; rm-ANOVA ‘genotype’ F (1, 22) = 6.277; *P* = 0.020). Similarly, faster recovery of RotaRod running was observed with Nestin-Grn-OE versus STOP-Grn^flfl^ mice (Additional file 1: Figure S3g). Tamoxifen per se had no effect in C57BL6 mice (Additional file 1: Figure S1b).

Voluntary Wheel Running (VWR) in the Phenomaster (Fig. 7b) is a test for coordination and endurance. At baseline running times and 24 h-rotations of Grn^−/−^ and Grn^+/+^ mice did not differ and they had no preference for running in either direction. After sciatic nerve injury, VWR dropped 1 day after the injury in both genotypes. The controls rapidly recovered and had no side preference. Grn^−/−^ mice also recovered runs and left turns, but right turns remained reduced, resulting in a reduction of the right/left ratio (Fig. 7b, right; *t*-test for AUCs 2-sided, unpaired, *P* = 0.041), likely reflecting lasting compensatory defects.

In the Tail Suspension Test (TST) (Fig. 7c) struggling times that are a measure of muscle strength and effort decreased after nerve injury, reflecting the motor deficit and possibly depression. This decrease was stronger in Grn^−/−^ mice (rm-ANOVA “genotype” F (1, 15) = 6.856 0.019) whereas SLICK-Grn-OE mice did not differ from the controls (not shown).

We used an over-ground gangway-walking task in the Motorater (Fig. 7d) to further assess subtle changes of body posture, locomotion and coordination after nerve injury. The height of the iliac crest dropped in SLICK-Grn control mice but remained at normal baseline level in tamoxifen treated SLICK-Grn-OE littermates (rm-ANOVA ‘genotype’ F = 6.096, df = 1, *P* = 0.0388 and interaction ‘genotype X time’ F = 2.550, df = 4 *P* = 0.0582). Both genotypes showed an increase and subsequent time-dependent recovery of the stride width i.e. the distance between both hind paws, without differences between genotypes. There was also no difference of front paw distances (not shown). The mice developed disturbances of coordination that manifested in changes of the ratios of the stride diagonals i.e. the distance between left hind and right front paw and vice versa. Normally, the distances are equal and the ratio is 1. In nerve injured mice, the LH-RF distance i.e. the diagonal of the injured hind paw to the contralateral front paw decreased and the opposite RH-LF increased resulting in a ratio <1. Analysis of the ratios for the strides of mid length (50 % quantile) revealed a strong increase of the variability after nerve injury that normalized in the tamoxifen treated group but not in the controls (rm-ANOVA ‘genotype’ and interaction ‘genotype X time’ *P* = 0.8637 and *P* = 0.5119). The ratios for the long strides (upper 10 % quantile) differed significantly between groups. In the SLICK-Grn-OE mice coordination recovered whereas this recovery was not achieved in the controls (rm-ANOVA ‘genotype’ F = 14.65, df = 1, *P* = 0.0050 and interaction ‘genotype X time’ F = 3.035, df = 4, *P* = 0.0314). The behavioural data suggested that progranulin promoted recovery of motor functions and coordination whereas progranulin deficiency delayed and impaired recovery.

### Recovery of sensory functions after sciatic nerve injury

Von Frey hair testing of touch sensitivity was used to assess sensory disturbances after nerve injury (Additional file 1: Figure S5). After the crush injury of the sciatic nerve, mice show mechanical hypersensitivity reflected by a left-shift of the von Frey response curve. SLICK-Grn-OE and SLICK-Grn mice showed similar left-shifts at day 1 and 3 after the injury, but in tamoxifen treated OE mice, the response curves and EC50 values returned to the pre-injury state within 7–14 days whereas control mice recovered normal responses only after 3 weeks (statistics for EC50 in Additional file 1: Figure S5). Hence, progranulin overexpression also improved recovery of sensory functions.

## Discussion

The present study shows that neuron specific overexpression of progranulin in mice promotes axonal regrowth, target re-innervation and motor function recovery after peripheral nerve injury. Oppositely, progranulin deficiency delayed regrowth, increased motor neuron deaths and increased pro-inflammatory microglia in the ventral horn. Together, these gain-of-function and loss-of-function experiments suggest that progranulin helps motor neurons to survive and to rebuild their axons and synapses.

The observed increase and acceleration of PKG1 immunofluorescence in the nerve suggests that the positive outcome was mediated by faster recovery of axonal transport, fiber regrowth and possibly pathfinding of re-growing axons, because PKG1 is crucial for pathfinding of peripheral neurons during development [36, 37]. PKG1 was used as a marker in the present study and as far as known, there is no direct functional link to progranulin, which likely is a substrate of axonal transport itself. At the site of the nerve lesion, progranulin was not only expressed in neuronal fibers but also by infiltrating immune cells, which may contribute to the extracellular pool of progranulin.

The observed enhancement of motor neuron regeneration in vivo agrees with previously observed neurotrophic functions of progranulin in primary neurons in vitro [8, 38]. As far as presently known, the neurotrophic effect is neither mediated through Trk-A (Ntrk1 = NGF receptor) nor does it require its transporter, sortilin [16, 17], which also acts as transporter for progranulin [13]. Recent high-throughput affinity chromatography analyses of protein interactomes did not retrieve sortilin as a progranulin binding partner but suggest interactions of progranulin with EGFR and with soluble Notch ligands [21, 22] pointing to EGF domain rich proteins, and possibly Notch as putative progranulin targets. This idea is supported by the 3D structural similarity between individual granulins and EGF motifs [19] and by the results of the present study.

Using a 2-step approach, first unbiased proteomics and subsequent Notch-focused analyses, we provide some evidence that progranulin indeed binds to and activates Notch receptors, which would agree with a number of progranulin’s effects e.g. on cancer growth, immune modulation, neurogenesis and neuron survival. Overexpression of progranulin was associated with upregulation of proteins involved in “regulation of transcription” and the transcription factor prediction of these upregulated proteins pointed towards signaling through Notch, Rbp-jkappa or Notch-dependent pathways. Indeed, we found an increase of Notch-dependent genes, Hes and Hey in the DRGs in progranulin overexpressing mice, again suggesting an enhancement of Notch-dependent gene transcription under these conditions.

Notch is normally activated by trans-binding of the canonical cell-bound Notch ligands, Jagged and Delta-like, presented by an opposing cell [39], whereas cis-binding of ligands presented by the Notch-bearing cell itself normally trigger Notch endocytosis and subsequent downregulation [40]. Our results show that progranulin binds to the NECD and increases mRNA levels of Notch target genes suggesting that progranulin promotes Notch processing and signaling. Two scenarios are conceivable: (i) binding of progranulin may elicit the canonical activation of Notch via sequential ADAM-γ-secretase cleavage at the plasma membrane, a process supposed to require cell-bound ligands [41] or (ii) binding of progranulin to the NECD may trigger Notch endocytosis, and subsequent processing of the NICD at the endosomal or lysosomal membrane [33, 34, 42]. The colocalization of progranulin with NECD at the cell surface, and with full-length Notch1 in intracellular vesicular structures favors the latter scenario but further studies are needed to dissect out the designation of vesicles that contain both proteins. Processing and cleavage of the NICD at the endosomal membrane is normally referred to as “ligand-independent” Notch processing because this signaling of Notch from the endosome occurs without binding of one of the canonical ligands [43, 44]. Endocytosis of progranulin is supposed to occur via the VPS10 beta propeller protein, sortilin [13, 45], and possibly related transporters, but a number of functions of progranulin are independent of sortilin [16] and unaffected in sortilin-deficient zebrafish [17]. Hence, additional routes may contribute to progranulin reuptake. Our proteomic data suggest that progranulin may have an active role in endocytosis, because a number of epsins including Eps8, Eps15 and Epn1 and ADP ribosylation factors including Arhgef2, Arhgef10 and Arhgef12, which are involved in receptor-triggered endocytosis [46, 47] were upregulated in SLICK-Grn-OE mice suggesting an adaptation to a higher demand or turn-over of this process. The GO term “transporting ATPase” was one of the significantly enriched GO terms. Oppositely, expression of Notch receptors and targets was mildly reduced in progranulin deficient mice suggesting that progranulin was needed to maintain Notch signaling.

Hence, our data point to a progranulin-mediated gain of Notch signaling. Thereby, progranulin may enhance and prolong the transcriptional regeneration program of injured neurons, which is switched on after axonal injury [48]. This program also involves surrounding Schwann cells and infiltrating immune cells, which all express Notch receptors [49, 50]. The progranulin-evoked initial activation of Notch signaling might be followed by Notch downregulation. This idea is lent from the cis-ligands i.e. those sent by the receiving cell itself. They also cause Notch endocytosis but this is followed by Notch degradation or downregulation and is inhibitory in nature [42, 51, 52]. Hence possibly, effects of progranulin are not unidirectional but time and context dependent, a phenomenon which has also been suggested for other soluble Notch ligands [53].

A number of interactors of progranulin have been identified, but still it is kind of an orphan ligand. Part of its encounters mark its travelling path from secretion, extracellular life, re-uptake and degradation. One of these previously described extracellular binding partners is perlecan [54], which promotes the growth of presynaptic neuromuscular boutons likely via bidirectional regulation of WNT and downstream β-catenin signaling [55], and hence may be relevant in the context of the present study. Our proteomic data did not point to WNT signaling but β-catenin was one of the regulated hits and may link WNT to Notch [56]. Progranulin was also described as an inhibitor of TNF-alpha receptors [14, 57] but this would not explain the pro-regenerative functions observed in the present study, and a direct interaction with TNFR was not unequivocally confirmed [15]. In addition, TNFR signaling may positively contribute to synapse stability and remodeling in axon-injured neurons [58] so that its antagonism cannot be conclusively linked with motor neuron regeneration and motor function recovery, but TNFR inhibition may contribute to the antinociceptive properties of progranulin [8] in models of TNFα-dependent neuropathic pain [59–61].

Progranulin has been mainly investigated in the context of neurodegenerative diseases of the central nervous system [62, 63]. A hallmark of such neurodegenerative diseases is an accumulation of pathological proteins and protein aggregates that were found to be more effectively cleared from the CNS in the presence of progranulin [64–67]. Removal of debris is a prerequisite for regeneration [68], and the subsequent restorations of neuromuscular junctions depends on muscle activity and proliferation of terminal Schwann cells and their communication with the growing axons [69–71]. This is regulated by secretory proteins, in part via exosomes [72–74], predestinating progranulin to be a messenger in this process. It is unknown though how exactly the message would be transferred, possibly via Notch as suggested by our results. The observed increase of Erk phosphorylation and Hey1 upregulation after stimulation with recombinant progranulin agrees with this hypothesis, but further neurotrophic receptors may be involved.

## Conclusion

In summary, we show that progranulin enhances and accelerates fiber regrowth and target re-innervation after sciatic nerve injury, likely mediated or contributed by binding to and activation of Notch signaling. As a result, progranulin’s overexpression was associated with faster recovery of sensory and motor functions in behaving mice showing the clinical relevance.

## Methods

### Generation of tamoxifen-inducible neuron-specific progranulin overexpressing mice

The generation of progranulin transgenic mice was done in collaboration with Ozgene Pty. Ltd. The plasmid 641_FSniper-pA was used for construction of the targeting vector consisting in an UbiC promoter was in front of a loxP flanked STOP cassette, which was followed by mouse progranulin cDNA and a phosphoglycerine kinase (PGK) Neo cassette, the latter flanked with FRT sites. The targeting vector was inserted into the Rosa26 locus by homologous recombination. The targeting vector was linearized and electroporated into Bruce4 embryonic stem cells derived from C57BL/6 mice [75]. Positive ES cells were identified by Southern blotting with 5’ (5Pii) and 3’ probes (P3) and 3 positive clones were injected into Balb/C blastocysts. Chimeras were identified and crossed with C57BL/6 J mice to obtain the germline-transmitted heterozygous floxed mice. The PGK-Neo-SD-IS cassette was removed by mating heterozygous mice with FlpE mice. Mice were then bred to homozygosity of the floxed allele (STOP-Grn^fl/fl^) and to remove Flp. A PCR based genotyping method was developed to detect the wild type (Primer: left: cgt gtt cgt gca agt tga gt and right: ggc gga tca caa gca ata at, WT 747 bp) and the floxed allele (left: gga tct gac atg gta agt aag c and right: gcc atc acc aca aga cac ac, floxed allel 425 bp).

Mice showing tamoxifen-inducible overexpression of progranulin in the majority of neurons were generated via Cre-loxP-mediated recombination by mating mice carrying the floxed STOP-Grn allele (STOP-Grn^flfl^) with a mouse line, the so-called SLICK-H Cre mice [27] enable specific gene recombination in the majority of central and about 80 % of peripheral neurons. They are on a C57BL6 genetic background. Successful Cre-recombinase mediated excision of the floxed STOP sequence was confirmed by genotyping of the floxed allele (removal of the 425 bp band) and by genotyping of Cre-recombinase as described [76] (Cre-Primer left: gaa agc agc cat gtc caa ttt act gac cgt ac; right: gcg cgc ctg aag ata tag aag a).

Pan-neuronal progranulin overexpressing mice were generated by mating Nestin-Cre mice with mice carrying the floxed STOP-Grn allele (STOP-Grn^flfl^). Nestin-mediated recombination commences around emryogenic day E11 and does not require tamoxifen. These mice were used in some experiments to eliminate tamoxifen as putative confounding factor or to get cultures of embryonic primary neurons. Key experiments of SLICK-Grn-OE mice were replicated with Nestin-Grn-OE mice.

Male and female 8–16 weeks old SLICK-Grn mice were treated with tamoxifen or vehicle to induce progranulin overexpression (SLICK-Grn-OE versus SLICK-Grn). The tamoxifen protocol consisted in once daily intraperitoneal injection of 0.15 mg/g of body weight for 5 consecutive days in 9:1 corn oil/ethanol followed by a free interval of 14–21 day before start of the experiment. Alternatively, mice received 4 weeks of tamoxifen feeding using commercially available tamoxifen pellets (Harlan, Germany) according to the manufacturer’s instructions, followed by a free interval of 1–2 weeks. The latter protocol was used for behavioral studies, long-term experiments and proteomics because it was better tolerated. Mostly, experiments started 2 weeks after finalizing tamoxifen feeding and after full recovery of the body weight. The tamoxifen pellets contain 5 % sucrose to increase palatability but still mice prefer normal pellets and may lose up to 5 % of their body weight during a 4-week feeding course, mostly during the first week with subsequent recovery. Tissue for proteome analysis was obtained 4–5 months after the last tamoxifen treatment. Treatment with i.p. tamoxifen for 5 days also causes a drop of the body weight up to 10 %, which is followed by recovery within 2 weeks.

For progranulin knockout mice (Grn^−/−^) [77], which are on a C57BL/6 J background we used sex and age matched C57BL/6 J mice or STOP-Grn^flfl^ as controls, the latter in experiments involving Grn^−/−^ and OE animals. All mice (floxed, all Cre and knockouts) were on a C57BL6/J genetic background.

For the preparation of E21 primary neuron cultures with progranulin overexpression, SLICK-Grn-OE mothers were treated with intraperitoneal tamoxifen (0.15 mg/g once daily) in the late phase of pregnancy (E17-E19). For the preparation of neuron cultures from pups, mothers were treated from E18 to P2. Pups get the tamoxifen through the mother’s milk. For early embryonic neuronal cultures, we used Nestin-Grn-OE mice.

Mice had free access to food and water and were maintained in climate controlled rooms at a 12-h light-dark cycle. The experiments followed the “Principles of laboratory animal care” (NIH publication No. 86–23, revised 1985), were approved by the local Ethics Committee for animal research (Darmstadt, Germany), adhered to the guidelines for pain research in conscious animals of the International Association for the Study of PAIN (IASP) and were in line with the European and German regulations for animal research.

### Injury of the sciatic nerve

Surgery was performed under 1.5–2 % isoflurane anesthesia with local lidocaine anesthesia of the skin. To induce a crush injury of the sciatic nerve that allows for fiber regrowth and regeneration a blunt Mayo-Hegar needle holder grabbed the sciatic nerve proximal of its separation and crushed the nerve for 30 s.

### Quantitative RT-PCR (QRT-PCR)

Total RNA was extracted from homogenized tissue with the RNAeasy tissue Mini Kit (Qiagen), and reverse transcribed using poly-dT as a primer to obtain cDNA fragments. QRT-PCR was performed using an ABI prism 7700 TaqMan thermal cycler (64 °C for 30 cycles) (Applied Biosystems) with the SybrGreen detection system and 2 primer sets for progranulin: pair-1 forward: 5’-CTAGATGGCTCCTGCCAGAC-3’; reverse: 5’-GCCATCACCACAAGACACAC-3’ and pair-2: forward: 5’-CCGAGGGTACCCACTACTCA-3’; reverse: 5’-GCCACAGCCTTCTTTCCATA-3’. Transcript regulation was calculated using the relative standard curve method with β-actin as housekeeping gene according to the manufacturer’s instructions.

For QRT-PCR of Notch receptors, ligands and target genes we used the primer pairs described in [78]. The PCR conditions were optimized for each primer set and normalized on the house-keeping genes ribosomal protein S13 (Rps13) and RNA-polymerase II (RNA-PolII).

The comparative mRNA quantification method was used as recommended for TaqMan analysis. The Ct values were normalized to the Ct of house-keeping genes and then the ΔCt levels were normalized on the reference group giving the ΔΔCt, which were Z-transformed for each gene individually and presented as heat maps.

### Transient cotransfection and coimmunoprecipitation

Transient transfection of HEK 293 EBNA cells was performed with Lipofectamin 2000 (Invitrogen, Karlsruhe, Germany) according to the manufacturer’s instructions. Plasmids were used as described previously [8, 53] or purchased from GeneCopoeia (pReceiver backbone with different C-terminal tags e.g. pReciever-M45 and -M46, and Lv202 or newly cloned). Plasmids are listed in Additional file 2: Table S3.

HEK 293 EBNA cells were lysed in ice-cold lysis buffer containing protease and phosphatase inhibitors. Following centrifugation at 16,200 g for 10 min, supernatants were subjected to immunoprecipitation in the presence of 50 μl of Immunosorb A (Medicago, Uppsala, Sweden) overnight at 4 °C. The following day, the beads were washed 3× in lysis buffer and the bead pellet was resuspended in 20 μl 4X Laemmli buffer, boiled (5 min 95 °C), and immune complexes were loaded onto SDS-polyacrylamide gels and transferred to nitrocellulose membranes. Membranes were blocked in 10 % blocking solution (Rotiblock, Roth, Karlsruhe, Germany) at RT for 1 h. All primary antibody incubations were performed in blocking buffer for 2 h at RT followed by incubation with fluorescent-labeled anti-mouse antibody for 1 h at RT. Immunocomplexes were visualized by fluorescence using Odyssey (LI-COR) according to the manufacturer’s instruction.

### Western blotting and ELISA

Protein extracts were prepared in RIPA lysis buffer or PhosphoSafe Buffer (Sigma) containing a protease inhibitor cocktail and PMSF 10 μg/ml, separated on 10, 12 or 14 % SDS-PAGE gels (30 μg/lane) and transferred to nitrocellulose membranes (Amersham) by semi-dry blotting. Unspecific binding sites were blocked with 3 % skimmed milk in PBS containing 0.5 % Tween 20 or in Odyssee buffer. Blots were incubated with primary antibodies (4 °C overnight) and subsequently with secondary antibodies in blocking buffer conjugated with IRDyes 680 or 800 (1:10,000 for 2–3 h at RT; LI-COR Biosciences). Blots were visualized and analyzed on the Odyssey Infrared Imaging System (LI-COR Biosciences). For total cell lysates β-tubulin was used as loading control.

Progranulin protein expression in tissue and in body fluids was analyzed with a progranulin enzyme immune assay (R&D systems) according to instructions of the manufacturer. Tissue pieces were homogenized and crude protein extracts prepared in PhosphoSafe Buffer containing Pefablock SC-protease-inhibitor mix. Samples were roughly adjusted to a protein concentration of 100 μg/ml (depending on the tissue, this was a dilution 1:5 (DRGs)-1:25 (Cortex)), and 100 μl were subjected to the ELISA. Cell culture supernatants were diluted 1:25, plasma samples 1:750, and each 100 μl were subjected to the ELISA. The ELISA was performed according to the instructions. The absorbance was read on an ELISA reader (SPECTRAFluor Plus) at 450 nm. Protein concentrations were determined with the Bradford method and progranulin concentrations were normalized to the measured protein level of the respective tissue sample and expressed as ng/mg protein, or are expressed as μg/ml or ng/ml for plasma and culture supernatants.

### Immunofluorescence and in situ hybridization

Mice were terminally anaesthetized with isoflurane and cardially perfused with cold 0.9 % NaCl, followed by 4 % paraformaldehyde (PFA) in 1× PBS for fixation. Tissues were excised, postfixed in 4 % PFA for 2 h, cryoprotected overnight in 20 % sucrose at 4 °C, embedded in tissue molds in cryomedium and cut on a cryotome or a vibratome (DRGs and sciatic nerve 10 or 12 μm, spinal cord 12 μm, brain 14 μm).

Slides were air-dried and stored at −80 °C. After thawing, slides were immersed and permeabilized in 1× PBS with 0.1 % Triton-X-100 (PBST), then blocked with 1 % blocking reagent (Roche), 3 % BSA or with 10 % donkey serum in PBST, subsequently incubated overnight with the first primary antibody in PBST at 4 °C, washed and incubated with the secondary antibody for 2–4 h at room temperature. The procedure was repeated for the second primary/secondary antibody pair, followed by 30 min incubation with 1 μg/ml DAPI (in some experiments) and embedding in Fluoromount (eBioscience). The general settings were optimized for the respective antibodies and tissues. Primary antibodies are listed in Additional file 2: Table S2. Secondary antibodies were labelled with fluorochromes including Cy3, Cy5, FITC and Alexa-dyes in green, red and blue (Sigma, Invitrogen, Chemicon).

For in situ hybridization mice were only briefly fixed, and fresh frozen tissue was cut at 14 or 18 μm on a cryotome. Air dried slides were fixed for 20 min in 4 % PFA in 1× PBS and acetylated. Sense and antisense riboprobes for mouse progranulin (nucleotides 55–692, length 637) were obtained by cloning PCR products into the pCR4 TOPO sequencing vector (Invitrogen) and subsequent in vitro transcription and labelling with digoxigenin (Dig-labeling kit, Roche). Sections were prehybridized for 2 h at 65 °C and hybridized at 70 °C for 16 h with 200 ng/ml of sense and antisense probes in the prehybridization mix (50 % formamide, 5 × SSC, 5 × Denhardt’s solution, 500 μg/ml herring sperm DNA, 250 μg/ml yeast tRNA) [29], washed in 0.2 % SSC at 65 °C and incubated with anti-Dig-AP in 0.12 M maleic acid buffer with 0.15 M NaCl, pH 7.5 and 1 % blocking reagent, washed in TBS, equilibrated in alkaline buffer (0.1 M Tris-HCl, 0.1 M NaCl, 0.05 M MgCl2, pH 9.5, 2 mM levamisole) and developed with BM Purple AP substrate (Roche Diagnostics). Slides were embedded in glycerol/gelatine or processed for post-in situ immunohistochemistry and analyzed on an inverted fluorescence microscope (AxioImager Z1, Zeiss, Germany).

### Quantification of immunofluorescent images

Tiled images were captured to cover the complete ventral horn, motor cortex or sciatic nerves or the muscle section. Images were stitched (Photoshop CS6) and analyzed by counting objects manually or by analyzing pixel intensities with help of FIJI (ImageJ). For “objects”, the readouts were the total number of ATF3 positive neurons in the ventral horn or the total number of α-Bungarotoxin positive neuromuscular synapses in a muscle section. For each mouse, a minimum of 5 non-overlapping images of >3 mice per group were analyzed. NMJs in a muscle section were marked, and then counted as objects by FIJI ImageJ. Overall, 30–40 non-overlapping muscle sections per time point, per genotype were analyzed. For nerves, all sections were stained and analyzed. To analyze the velocity of nerve regeneration and glia activation we used ImageJ to quantify pixel intensities. Each channel of an image was converted to 8bit grey scale, background subtracted, and the intensity threshold was automatically set with minor corrections. Intensities were then converted to rainbow pseudocolor for visualization. Line or rectangular ROI intensity profiles were plotted and results exported to SigmaPlot 12.5, smoothed using the Loess smoothing function and AUCs (Intensity x distance) calculated using the linear trapezoidal rule. AUCs were subsequently used for statistical comparisons. In addition, we measured the area of the lesion site and used the mean pixel intensity inside of the lesion area including the proximal border for statistical comparisons of fiber density.

### Behavioral assessment of locomotion and sensory functions

All tests were performed by an investigator who was unaware of the mouse genotype or treatment and included 8–12 mice per group and test. Locomotion was assessed with an accelerating RotaRod (UgoBasile), the Tail Suspension Test, a gangway walking task in the MotorRater (TSE, Germany) and computerized voluntary wheel running in home cage conditions in a Phenomaster (TSE). Before starting experiments mice were habituated for three consecutive days to the test room, test cages and apparatuses. Habituation to the RotaRod test encompassed 2–3 training sessions each day. For the MotoRater 3–5 training sessions were used. In the Phenomaster mice were adapted to the drinking bottles for 5 days in their home cage and were then adapted to the Phenomaster cage for 24 h before starting a 24 h test period.

The settings for the accelerating RotaRod test were a start speed of 15 rpm, maximum 30 rpm, ramp 3 rpm/min, cut-off time 6 min. The fall-off time was averaged from 2 tests.

The Motorater allows for quantitative complex kinematic evaluation of the spontaneous locomotion of the test mouse [79]. We used ‘over-ground walking’ as the primary task, in which mice had to walk in one direction in a Plexiglas gangway with a length of 150 cm, a width of 5 cm and a wall height of 15 cm, with two mirrors on each side. Locomotion was captured with a high-speed camera positioned underneath the gangway. A second camera recognizes the movements and allows for automatic tracking of the mouse. The automated recording was set for a walking distance of 40 cm (the middle part of the runway) and analyzed with a rodent-adapted version (TSE) of Simi Motion (simi reality motion systems, Germany). The software provides an extensive platform for motion capture and 2D/3D movement analysis. It enables automatic detection of markers on the skin overlying important anatomical landmarks including the tail, feet and ankles, knee and iliac crest, which were painted onto the skin directly before the test. Distances and angles between these markers and the floor were calculated with help of the software.

For analysis of locomotor ability, we chose a set of the most relevant parameters, i.e. distances between the front and hind paws, diagonal distances between front and hind paws and height of the iliac crest that tends to drop after nerve injury and can be interpreted as a measure for hip muscle strength. To assess coordination, we calculated ratios of the basic distances of the injured versus uninjured side including “injured left hind paw to the right front paw” versus “right hind paw to left front paw” (LH-RF/RH-LF). For each mouse and test run we calculated for each parameter the mean, the lower 10 % and upper 10 % quantiles from the individual steps to assess the mean, minimum and maximum step lengths or distances. Each run provided on average 245 measurements for each parameter (range 50–1234).

The Tail Suspension test (TST) is often used for the evaluation of depression-like behavior but it is also a readout for struggling and locomotor ability. The mice were suspended by the tail from a hook approximately 30 cm above the floor. The tail was fixed with adhesive tape to the hook. The behavior consisting in struggling and immobile hanging from the hook was observed and captured with a camera positioned above the testing apparatus. Each test lasted 5 min. The time of struggling and the relative decrease of struggling after nerve injury were considered as indicators muscle weakness and depression resulting from an unsolvable and aversive situation [80]. All behavioral tests were performed at baseline and repeatedly up to 6 weeks after sciatic nerve injury.

The Phenomaster (TSE) offers an automated metabolic and behavioral monitoring in home cage environments. Drinking and feeding behavior and running behavior (running time, left- and right sided rotations), were monitored with high-precision weight sensors for liquid and food dispensers, and with a PC-controlled running wheel. Mice had free access to water and standard diet pellets and free access to voluntary running wheel during adaptation and 24 test periods. Mice can choose to enter the wheel by turning left or right. The number of left and right entries was counted and ratios considered as a measure of coordination and side preference.

To assess sensory functions a standard set of von Frey hairs (Ugo Basile) was used. Each hair was applied perpendicularly 10 times to the plantar side of the left ipsilateral or the contralateral right hind paw and the number of withdrawal reactions was counted and plotted as withdrawal frequency (in %) versus the stimulation force of the respective von Frey Hairs, giving the response curves. The slope and EC50 values of the von Frey response curves were calculated with a standard sigmoidal Emax model.

### Culture of primary neurons and human embryonic kidney cells

Primary neuron-enriched cultures were prepared from E21 mouse embryos by dissecting cortices into HBSS (Ham’s balanced salt solution, Dulbecco) and 10 mM HEPES, followed by digestion with 5 mg/ml collagenase A and 1 mg/ml dispase II (Roche Diagnostics, Mannheim, Germany) prior to treatment with 0.25 % trypsin (GibcoBRL, Karlsruhe, Germany). Alternatively, primary neuron cultures were prepared from mouse or rat dorsal root ganglia from adult animals. Triturated cells were centrifuged through a 15 % BSA solution, resuspended, counted and adjusted to the required density and plated on poly-L-lysine and laminin coated 6-well plates (~10 exp5 per well), or on cover slips (~0.5x10 exp3 per cover slip) in Neurobasal medium (GibcoBRL) containing 2 % (vol/vol) B27 supplement (GibcoBRL), 10 μM Ara-C, 50 μg/ml Pen-Strep, 100 ng/ml NGF and 200 mM L-glutamine with half exchange of the medium every 3 days. The medium for adult neurons was without AraC and Pen-Strep.

Human embryonic kidney, HEK293 cells were grown in 1:1 EMEM and Ham’s F12 medium, 15 % supplemental fetal bovine serum, 2 mM glutamine and 1 % nonessential amino acids (NEAA). All cells were kept in an incubator at 37 °C, 95 % humidity and 5 % CO_2_ atm. For Co-IPs sub-confluent HEK293 cells were transiently co-transfected with mammalian plasmid vectors with full-length human or mouse progranulin with/without C-terminal tag or with Flag or V5 tagged Notch cDNAs as described [8, 53] or purchased from GeneCopoeia (pReceiver backbone; list of vectors in Additional file 2: Table S3) Cells transfected with empty vectors (tag only) were used as controls. All cells were incubated at 37 °C, 5 % CO_2,_ 95 % humidity.

### Phospho-Erk

Primary rat DRG neuron cultures (500 cells per cover slip) were stimulated in serum-free medium with 10 ng/ml recombinant purified human progranulin (R&D Systems, 95 % purity, endotoxin <0.01 EU per 1 μg) for 8 h, starting 48 h after plating. Cells were rinsed in cold PBS, fixed with 4 % PFA for 20 min, permeabilized for 20 min with 0.1 % Triton-X-100 in PBS and blocked for 1 h using 3 % BSA in PBST. Incubations with primary and secondary antibodies and DAPI were applied sequentially without extensive washing to avoid loss of cells. The cells were embedded in Fluoromount (eBioscience). Anti-phopho-Erk immunostainings were quantified by software-aided (AutMess modul of AxioVision) counting of P-Erk positive neurons relative to all neurons (NF200 positive) in the image.

### Immunofluorescence analysis of notch—progranulin co-expression

Primary mouse DRG neuron cultures were prepared as described above. After 16 h in culture, primary DRG cells were washed and fixed in 4 % PFA in 1× PBS for 20 min. Cells were permeabilized in 1× PBS with 0.1 % Triton-X-100 (PBST) and blocked with 5 % BSA in PBST for 30 min. Blocking was followed by incubation with the primary antibodies (Additional file 2: Table S2) in 1 % PBST at 4 °C overnight. After washing, cells were incubated with the Cy3 or Alexa-488 labelled secondary antibodies for 2 h at room temperature together with 1 μg/ml DAPI. After final washing, cells were embedded in Poly/Mount (Polysciences, Inc.). Images were captured on a Leica TCS SP8 confocal laser scanning microscope equipped with Leica LAS X software.

The co-dependent or random nature of apparent colocalizations was tested using the ‘Intensity Correlation Analysis’ (ICA) that generates ICA plots and Intensity Correlation Coefficients (ICQ), and by using Pearson Correlation coefficients, R [81], Mander’s M1/M2 and Coste’s method and object based algorithms. The analysis is bundled with the JACoB plugin of FIJI ImageJ [35] and was performed with standard settings.

### Proteome analysis of the prefrontal cortex

To assess underlying molecular mechanisms, we performed a deep proteome analysis of the prefrontal cortex in aged Grn^+/+^, Grn^−/−^, SLICK-Grn-OE (TAM) and SLICK-Grn (noTAM) mice. Each 3 prefrontal cortices were rapidly excised and snap frozen in liquid nitrogen and stored at −80° until protein extraction. The mass spectrometry (MS) proteomics data has been deposited to the ProteomeXchange Consortium via the PRIDE partner repository with the dataset identifier PXD004087, along with a detailed description of the protein extraction and MS analysis.

### Gene ontology annotation and overrepresentation analysis

Proteomic data were analyzed with Perseus 1.5.2.6 [82]. Proteins were quality filtered according to unique peptides, sequence coverage, putative contaminants and a minimum of 3 valid values, and text filtering was used to exclude some highly abundant proteins. LFQ values were then LN transformed and missing/zero values were imputed from the normal distribution. Volcano plots were used to assess differences between 2 groups. The control groups (Grn^+/+^ and SLICK-GrnOE without tamoxifen) were similar and were combined.

The biological roles of the regulated proteins were identified by means of over-representation analysis (ORA) using the web-based GeneTrail tool (http://genetrail.bioinf.uni-sb.de/) [83]. This compared terms annotated to up- or downregulated proteins (GO; http://www.geneontology.org/) with the occurrence of terms among the set of all identified proteins (ORA parameters: *p*-value threshold *t*_*p*_ = 0.05 and Bonferroni α correction). The ontology annotations for “cellular component”, “biological process” and “molecular function” and KEGG pathways were included in the analysis.

Based on the GO results, which showed an enrichment of “transcription” and similar terms for the proteins, which were upregulated in SLICK-Grn-OE mice we submitted the set of upregulated proteins to a transcription factor prediction analysis using Tfacts (http://www.tfacts.org) [32]). The analysis is based on Chi-Square statistics to compare the presence of TF-regulated genes in a query set with the occurrence of genes regulated by the respective TF in the database. Significance was defined by *P* < 0.05, Bonferroni adjusted, and Random Control ≤ 3 %, which is the percentage by which a TF is called significant after random simulation of a gene list. Because the search predicted Rbpj (Recombination Signal Binding Protein for Immunoglobulin Kappa J Region) to be active, we retrieved genes with Rbpj motifs up to 500 bp upstream of the transcription start site from Motifmap (http://motifmap.ics.uci.edu/), compared the set of 507 entries with the set of upregulated proteins and with 10 sets of random gene selections. The frequency of positive hits was then compared with contingency tables and Chi Square statistics. Rbpj acts as a transcription repressor when it is not bound to Notch proteins and an activator when it is bound to Notch proteins.

The TF search also revealed an enrichment of basic helix loop helix transcription factor binding sites among the regulated proteins, which act downstream of Notch. The datasets of regulated proteins were therefore subsequently searched for proteins that have GO annotations for “Notch signaling pathway”, retrieved from AmiGO (http://amigo.geneontology.org/amigo/term/GO:0007219), are involved in Notch endocytosis or have been published as Notch target genes or interactors [84–86]. Sets of genes were compared using NetVenn (http://probes.pw.usda.gov/NetVenn/start.php) [87].

### Statistics

SPSS 22.0 was used for statistical evaluation. Data are presented as means ± SD. Behavioral data were analyzed using ANOVA for repeated measurements for time courses and one-way ANOVA or *t*-tests to compare areas under the curve. The latter were calculated according to the linear trapezoidal rule. Counts of neurons, qRT-PCR and immunoblot results were analyzed with Student’s *t*-tests (for two groups), one-way ANOVA or ANOVA for repeated measurements (rm-ANOVA). *P* was set at 0.05 for all statistical comparisons. In case of multiple comparisons, we used a correction of alpha according to Bonferroni or Dunnett versus control group. qRT-PCR results of DRGs were Z-transformed to allow for a comparison of genes with different expression levels, according to the formula Z = (X-μ)/SD, where X is the individual value, μ is the mean and SD is the standard deviation of all samples for the respective gene.

Statistics of immunofluorescence quantifications and proteomics are explained in the respective sections.

## Additional files

Additional file 1: Supplementary Figures. (DOCX 3067 kb)
Additional file 2: Supplementary Tables. (XLSX 27 kb)

## Abbreviations

EYFP: Enhanced green or yellow fluorescent protein
ANOVA: Analysis of variance
ATF3: Cyclic AMP-dependent transcription factor 3
AUC: Area under the curve
bHLH: Basic helix-loop-helix transcription factors
Co-IP: Co-immunoprecipitation
Cre: Cre-recombinase
DAVID: The Database for Annotation, Visualization and Integrated Discovery
DRG: Dorsal root ganglia
EGF: Epidermal growth factor
EGFL: Epidermal growth factor-like
FTLD: Frontotemporal lobar degeneration
GO: Gene ontology
Grn: Progranulin (official gene name)
IR: Immunoreactivity
NECD: Notch extracellular domain
NICD: Notch intracellular domain
NMJs: Neuromuscular junctions
OE: Overexpression
PKG1: Cyclic GMP dependent protein kinase type 1
Rbpj: Recombination Signal Binding Protein For Immunoglobulin Kappa J Region
SLICK: Single neuron labeling with Cre-inducible knockout
SV2: Synaptic vesicle protein
TAM: Tamoxifen
TF: Transcription factor
TNFα: Tumor necrosis factor alpha
VPS10: Vacuolar protein sorting ﻿10
VWR: Voluntary Wheel Running
Y2H: Yeast two hybrid
αBGX: Alpha bungarotoxin
