# Supplementary material for: Progranulin promotes peripheral nerve regeneration and reinnervation: role of notch signaling
Source: Mol Neurodegener. 2016 Oct 22;11:69. doi: 10.1186/s13024-016-0132-1 (PMC5075406; doi:10.1186/s13024-016-0132-1)
Supplement: Additional file 1: — Supplementary Figures. (DOCX 3067 kb) [file 13024_2016_132_MOESM1_ESM.docx]

# Supplementary Figures

# Suppl. Figure 1:


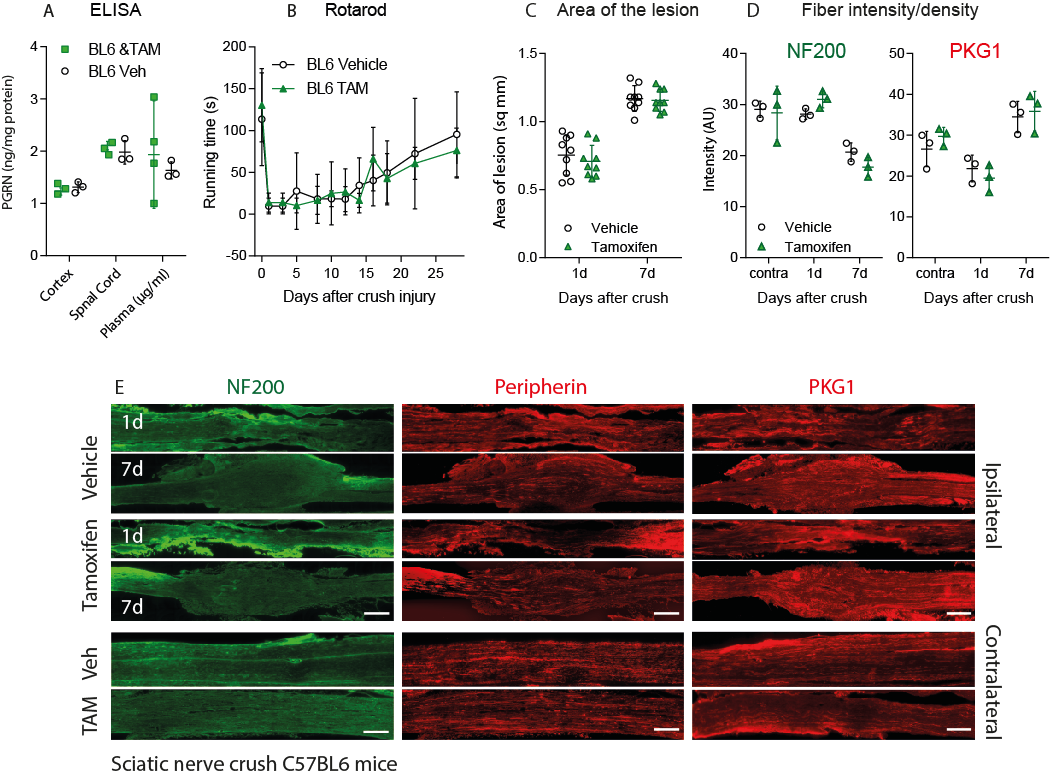


### Suppl. Figure 1:

Tamoxifen control experiments in C57BL6 mice: Tamoxifen per se did not alter progranulin protein expression in nervous tissue or plasma, did not alter the time course of crush-injury evoked motor function deficits in the RotaRod test and had no effects on sciatic nerve fiber morphology or regrowth.

**a:** Enzyme immunoassay of progranulin in cortex, spinal cord and plasma in wild type C57BL6 mice treated with vehicle or tamoxifen (0.15 mg/gram of body weight for 5 consecutive days in 9:1 corn oil:ethanol i.p., followed by free interval of 2 weeks before tissue dissection). The figure shows scatter plots of individual mice with mean ± SD.

**b:** Time courses of the running times on a constant speed RotaRod before and after crush injury of the sciatic nerve in C57BL6 mice treated with vehicle or tamoxifen (5 days i.p., free interval of 2 weeks before surgery). Running times did not differ between groups. The data show the mean ± SD of 8 mice per group; rm-ANOVA n.s..

**c:** Time course of the crush lesion size, which was determined in FIJI ImageJ on the basis of the morphology of immunofluorescent stainings. The increase of the area 7d after the injury is caused by swelling. The data are from 3 mice per group, each 3 stainings. There was no difference between vehicle and tamoxifen groups.

**d:** Time course of NF200 and PKG1 immunofluorescent fiber intensities inside of the lesion including the proximal border. There was no difference between vehicle and tamoxifen groups groups.

**e:** Exemplary immunofluorescence images of the ipsilateral and contralateral sciatic nerves after crush injury in C57BL6 mice treated with vehicle or tamoxifen (5 days i.p., free interval of 2 weeks before surgery). Representative result of n = 3 per group, per time point. Scale bars 500 µm.

# Suppl. Figure 2:


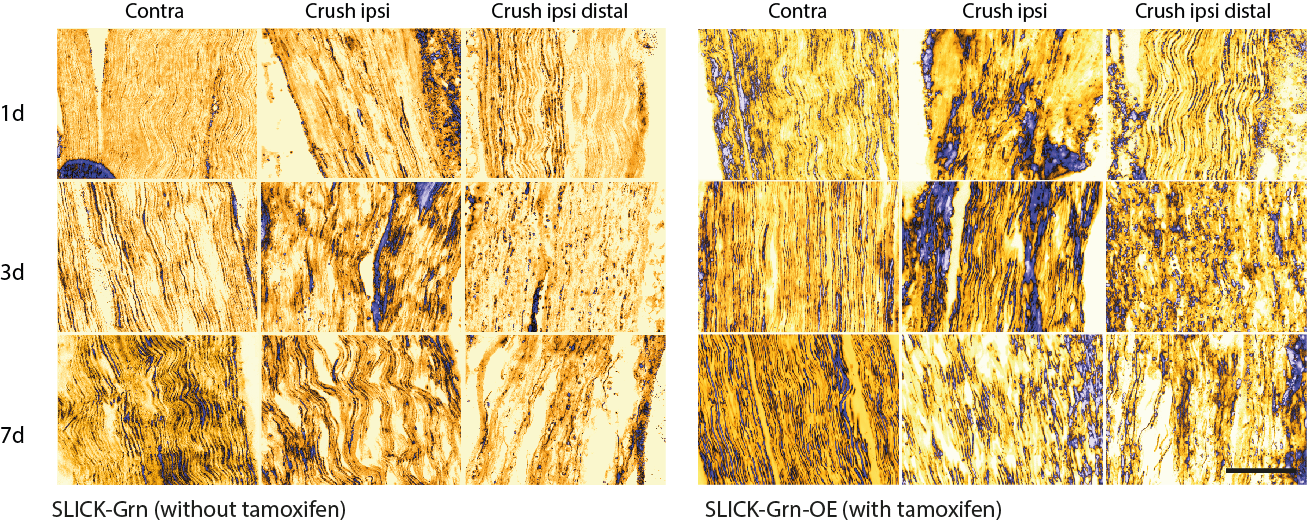


### Suppl. Figure 2:

Immunofluorescent analysis of progranulin expression in the sciatic nerve after crush injury. The images show pseudocolors (yellow – blue) of the immunofluorescent intensities 1, 3 and 7 days after crush injury at the lesion site and distal of it. The contralateral side is used as control. Progranulin immunoreactivity of nerve fibers and infiltrating immune cells appears in blue. Scale bar 200 µm.

# Suppl. Figure 3:


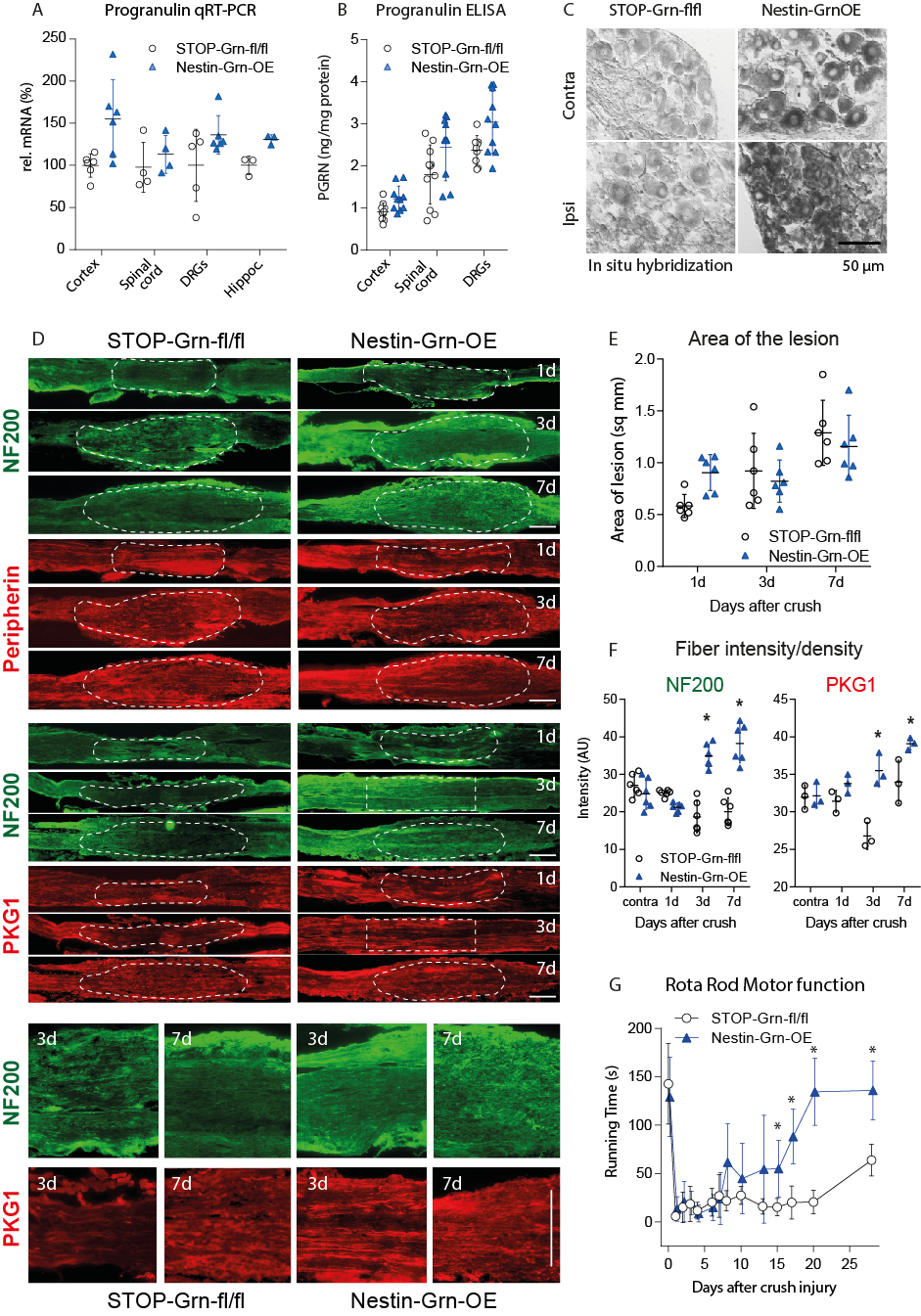


### Suppl. Figure 3:

Characterization of pan-neuronal Nestin-Cre mediated progranulin overexpressing mice.

Nestin-Grn-OE mice were generated by mating homozygous STOP-Grn^flfl^ mice (progranulin cDNA with leading STOP sequence inserted into Rosa26 locus) with Nestin-Cre mice, resulting in overexpression of progranulin in all neurons of the central and peripheral nervous system. The genetic background of all mice was C57BL6.

**a:** Quantitative RT-PCR of progranulin mRNA in cortex, spinal cord, dorsal root ganglia (DRGs), and hippocampus in STOP-Grn^flfl^ and Nestin-Grn-OE mice. Each symbol is a mouse, the line is the mean and whiskers show the standard deviation (SD).

**b:** Enzyme immunoassay of progranulin in cortex, spinal cord and DRGs in STOP-Grn^flfl^ and Nestin-Grn-OE mice. The figure shows scatter plots of individual mice with mean ± SD.

Nestin-Cre-mediated recombination significantly increased Grn mRNA and protein expression throughout regions (rm-ANOVAs for genotype P < 0.05).

**c:** In situ hybridization of progranulin mRNA in the DRGs ipsi- and contralateral of a sciatic nerve injury in STOP-Grn^flfl^ and Nestin-Grn-OE mice. Scale bars: 50 µm.

**d:** Immunofluorescence analyses of the ipsilateral sciatic nerves 1d, 3d and 7d after crush injury of the sciatic nerve in STOP-Grn^flfl^ and Nestin-Grn-OE mice. The upper panels show reconstructions from tiled images of approximately 4 mm of the sciatic nerve from proximal to distal of the lesion. The lower panels show higher magnifications of NF200 and PKG1 from the mid-lesion 3d and 7d after the injury. Comparison of the fiber structure and morphology suggests a faster progression through the stages of fiber regeneration in Nestin-Grn-OE mice. Hypo-dense or a fragmented morphology indicates fiber degradation. This is followed by clearing of fiber debris and start of regrowth, the latter indicated by stronger intensity at the proximal border with fibers outreaching into the lesion area. The images are representative reconstructions of n = 3 mice per group and time point. For quantification, the area as shown by the dotted lines was assessed on the basis of the morphology. Area, perimeter and mean intensities were analyzed with FIJI ImageJ. Scale bars: 500 µm.

**e:** Scatter plot of the areas of the lesions. Data are results of each 4 sections of n = 3 mice per group and time point. The apparent increase of the area at 3d and 7d in both groups is caused by the swelling of the nerve in the region of the injury. The areas did not differ between genotypes up to 7d after injury.

**f:** Mean fluorescent intensities of NF200 and PKG1 immunoreactive fibers inside of the lesion including the proximal border. Higher intensities in Nestin-Grn-OE mice suggests stronger or faster regrowth after the injury. Peripherin revealed reduced fragmentation in Nestin-Grn-OE but mean intensities did not differ between genotypes.

**d:** Time courses of the running times on a constant speed RotaRod before and after crush nerve injury of the sciatic nerve in STOP-Grn^flfl^ and Nestin-Grn-OE mice (mean ± SD; n=8). Asterisks show significant differences between genotypes (rm-ANOVA, subsequent t-tests of time points, * P < 0.05).

# Suppl. Figure 4:

#
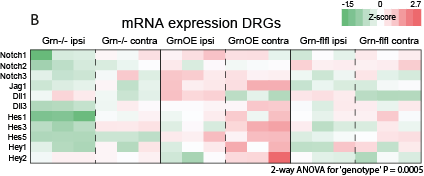

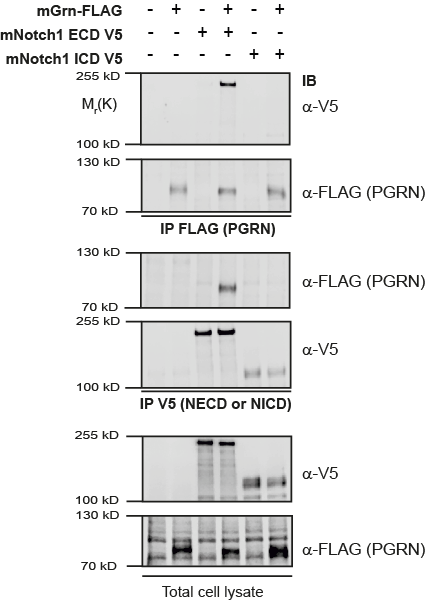


**4A:** Co-immunoprecipitation analysis of mouse progranulin (PGRN) with the extracellular or intracellular domains (NECD and NICD) of mouse Notch1, and reciprocally. HEK293 cells were transiently co-transfected with mNotch1-ECD-V5 or mNotch1-ICD-V5 and Flag-tagged full length mouse progranulin (mGrn-Flag) (all C-terminal tags). IPs were performed with anti-Flag and anti-V5 followed by immunoblot analysis of anti-V5 (for Notch) and anti-Flag (for progranulin, PGRN). Input blots (total cell lysate) were done with 1/10^th^ of the protein subjected to IP and are shown in the bottom.

**A**

**4B:** Heat maps of Z-cores of QRT-PCR mRNA levels of Notch receptors, Notch ligands and target genes in ipsilateral and contralateral L4 and L5 DRGs of Nestin-Grn-OE, Grn^-/-^ and STOP-Grn^flfl^ mice 7 days after sciatic nerve injury (n = 3 samples per genotype, each sample consisting in pooled L4 and L5 ipsilateral or contralateral DRGs of 3 mice, 3 replicate RT-PCR analyses of each sample for each gene). Two housekeeping genes, RNA Pol-III and Rps13, were used for calculation of ΔCt values. The ΔCt data of the respective genes were Z-transformed for each gene separately. Z-scores were color-coded ranging from dark green (low expression) to dark red (high expression). Hes levels tended to be lower ipsilateral versus contralateral in all genotypes, whereas Dll1 was increased ipsilateral in all genotypes. Z-transformed data were submitted to 2-way ANOVA for “gene” by “genotype” and groups differed significantly with P = 0.0005.

# Suppl. Figure 5:

#
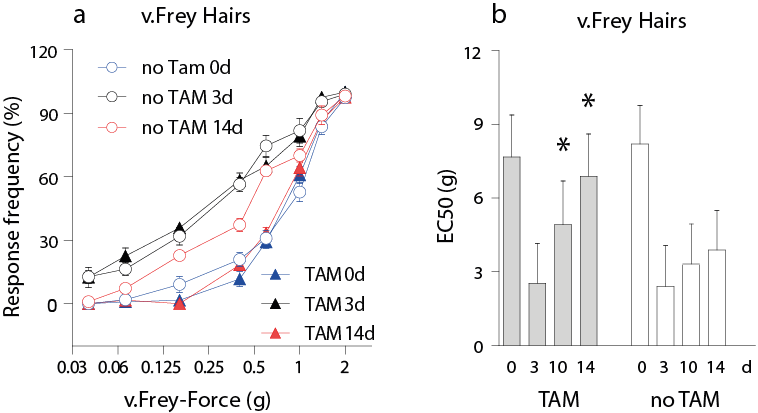


Suppl. Fig. 5: Nociception in SLICK-Grn-OE (TAM) and SLICK-Grn (noTAM) mice before and after nerve injury

**a:** Paw withdrawal curves on stimulation with graded strength von Frey hairs before and after crush injury of the sciatic nerve. 10 stimuli were applied with each hair and the number of withdrawals were counted and plotted versus the applied force. After crush nerve injury the response curves were shifted to the left in both groups showing nociceptive hypersensitivity, but in the SLICK-Grn-OE (TAM) group, the response curve returned faster to baseline showing a faster recovery of normal nociceptive sensitivity.

**b:** EC50 levels of von Frey responses in SLICK-Grn-OE (TAM) and SLICK-Grn (noTAM) mice before and after crush injury of the sciatic nerve. Data are the mean ± SD of 8 mice per group, * P < 0.05; rm-ANOVA, subsequent 2-tailed t-test between genotypes.

# Legend to Graphical Abstract

We show in the present manuscript that progranulin (PGRN) acts as a Notch ligand. It coimmunoprecipitates with all four Notch receptors. The binding site lies within the extracellular domain, NECD. It colocalizes with the NECD at the cell surface and with full-length Notch in intracellular vesicular structures suggesting endocytosis and possibly processing of Notch at the endosomal membrane [[1-3](#_ENREF_1)]. Overexpression of PGRN or stimulation with recombinant progranulin increases transcription of Notch-target genes, Hes and Hey, showing that PGRN enhances Notch signaling, which is involved in processes of cell renewal and differentiation. In line with this idea, neuronal PGRN overexpression in mice accelerated axonal regeneration after injury and rebuilding of neuromuscular junction (NMJs) and enhanced recovery of motor and sensory functions. Oppositely, PGRN deficiency increased motor neuron death and neuroinflammation.

Notch is normally activated by binding of trans-ligands, Jagged (Jag) and Delta-like (Dll), of the opposing cell eliciting a 2-step cleavage, mediated first by ADAM to cleave off the NECD and second, by γ-secretase to cleave off the NICD. The NECD is endocytosed together with the ligand by the signal-sending cell whereas the NICD translocates to the nucleus, where it forms a transcriptional complex with the DNA-binding protein Rbp-jκ, Mastermind (Mam) and transcriptional co-activators to release the repression of Notch target genes including the transcription factors Hes, Hey, Myc and HIF1alpha [[4](#_ENREF_4), [5](#_ENREF_5)]. In contrast, cis-interactions – between Notch and a ligand expressed in the same cell – trigger endocytosis and degradation and are inhibitory in nature [[6](#_ENREF_6), [7](#_ENREF_7)]. Progranulin is a secreted still orphan protein and likely acts as a soluble ligand contributing to the fine tuning of Notch signaling.

# References

1. Yamamoto S, Charng WL, Bellen HJ: **Endocytosis and intracellular trafficking of Notch and its ligands.** *Curr Top Dev Biol* 2010, **92:**165-200.

2. Kandachar V, Roegiers F: **Endocytosis and control of Notch signaling.** *Curr Opin Cell Biol* 2012, **24:**534-540.

3. Vaccari T, Lu H, Kanwar R, Fortini ME, Bilder D: **Endosomal entry regulates Notch receptor activation in Drosophila melanogaster.** *J Cell Biol* 2008, **180:**755-762.

4. Bray SJ: **Notch signalling: a simple pathway becomes complex.** *Nat Rev Mol Cell Biol* 2006, **7:**678-689.

5. Kopan R, Ilagan MX: **The canonical Notch signaling pathway: unfolding the activation mechanism.** *Cell* 2009, **137:**216-233.

6. Sprinzak D, Lakhanpal A, Lebon L, Santat LA, Fontes ME, Anderson GA, Garcia-Ojalvo J, Elowitz MB: **Cis-interactions between Notch and Delta generate mutually exclusive signalling states.** *Nature* 2010, **465:**86-90.

7. del Alamo D, Rouault H, Schweisguth F: **Mechanism and significance of cis-inhibition in Notch signalling.** *Curr Biol* 2011, **21:**R40-47.
